# Synthesis and Preclinical Evaluation of Novel ^68^Ga-Labeled (*R*)-Pyrrolidin-2-yl-boronic Acid-Based PET Tracers for Fibroblast Activation Protein-Targeted Cancer Imaging

**DOI:** 10.3390/ph16060798

**Published:** 2023-05-28

**Authors:** Shreya Bendre, Hsiou-Ting Kuo, Helen Merkens, Zhengxing Zhang, Antonio A. W. L. Wong, François Bénard, Kuo-Shyan Lin

**Affiliations:** 1 Department of Molecular Oncology, BC Cancer Research Institute, Vancouver, BC V5Z 1L3, Canada; sbendre@bccrc.ca (S.B.); hkuo@bccrc.ca (H.-T.K.); hmerkens@bccrc.ca (H.M.); zx1981ok@hotmail.com (Z.Z.); antwong@bccrc.ca (A.A.W.L.W.); fbenard@bccrc.ca (F.B.); 2 Department of Functional Imaging, BC Cancer Research Institute, Vancouver, BC V5Z 4E6, Canada; 3 Department of Radiology, University of British Columbia, Vancouver, BC V5Z 1M9, Canada

**Keywords:** fibroblast activation protein α (FAP-α), cancer-associated fibroblasts (CAFs), FAP inhibitors (FAPIs), PET imaging, gallium-68, (*R*)-pyrrolidin-2-yl-boronic acid-based radiopharmaceuticals

## Abstract

Fibroblast activation protein (FAP) is a membrane-tethered serine protease overexpressed in the reactive stromal fibroblasts of >90% human carcinomas, which makes it a promising target for developing radiopharmaceuticals for the imaging and therapy of carcinomas. Here, we synthesized two novel (*R*)-pyrrolidin-2-yl-boronic acid-based FAP-targeted ligands: SB02055 (DOTA-conjugated (*R*)-(1-((6-(3-(piperazin-1-yl)propoxy)quinoline-4-carbonyl)glycyl)pyrrolidin-2-yl)boronic acid) and SB04028 (DOTA-conjugated ((*R*)-1-((6-(3-(piperazin-1-yl)propoxy)quinoline-4-carbonyl)-D-alanyl)pyrrolidin-2-yl)boronic acid). ^nat^Ga- and ^68^Ga-complexes of both ligands were evaluated in preclinical studies and compared to previously reported ^nat^Ga/^68^Ga-complexed PNT6555. Enzymatic assays showed that FAP binding affinities (IC_50_) of ^nat^Ga-SB02055, ^nat^Ga-SB04028 and ^nat^Ga-PNT6555 were 0.41 ± 0.06, 13.9 ± 1.29 and 78.1 ± 4.59 nM, respectively. PET imaging and biodistribution studies in HEK293T:hFAP tumor-bearing mice showed that while [^68^Ga]Ga-SB02055 presented with a nominal tumor uptake (1.08 ± 0.37 %ID/g), [^68^Ga]Ga-SB04028 demonstrated clear tumor visualization with ~1.5-fold higher tumor uptake (10.1 ± 0.42 %ID/g) compared to [^68^Ga]Ga-PNT6555 (6.38 ± 0.45 %ID/g). High accumulation in the bladder indicated renal excretion of all three tracers. [^68^Ga]Ga-SB04028 displayed a low background level uptake in most normal organs, and comparable to [^68^Ga]Ga-PNT6555. However, since its tumor uptake was considerably higher than [^68^Ga]Ga-PNT6555, the corresponding tumor-to-organ uptake ratios for [^68^Ga]Ga-SB04028 were also significantly greater than [^68^Ga]Ga-PNT6555. Our data demonstrate that (*R*)-(((quinoline-4-carbonyl)-d-alanyl)pyrrolidin-2-yl)boronic acid is a promising pharmacophore for the design of FAP-targeted radiopharmaceuticals for cancer imaging and radioligand therapy.

## 1. Introduction

Cancer-associated fibroblasts (CAFs) found in the tumor microenvironment are functionally and phenotypically distinct from normal fibroblasts found in non-cancerous tissues [1]. Immuno-histochemical studies performed by Chesa et al. revealed that CAFs in several different primary and metastatic carcinomas, including colorectal, breast, ovarian, bladder, and lung carcinomas, overexpress a transmembrane glycoprotein called fibroblast activation protein (FAP) [2]. FAP is a cell-surface serine hydrolase shown to have dipeptidyl exopeptidase and endopeptidase activities [3]. FAP has a restricted normal tissue distribution including normal fibroblasts, but is overexpressed in the reactive stromal fibroblasts of >90% of human epithelial carcinomas (breast, lung, colorectal etc.) [2,3,4,5]. Moreover, its enzymatic activity is considered crucial for the promotion of tumor growth [6]. FAP’s constrained expression combined with its role in invasion and tumor metastasis, makes it a promising target for developing FAP-targeted radiopharmaceuticals for the imaging and therapy of carcinomas. In this regard, several different strategies including the use of antibodies [7,8], CAR T-cells [9,10], and FAP vaccines [11], and most significantly small-molecule inhibitors [6,12,13,14,15], have been explored for FAP targeting.

The two most noteworthy groups exploited for the development of small-molecule based FAP inhibitors are either 2-cyanopyrrolidine derivatives or 2-pyrrolidinylboronic acid derivatives (boroPro). Additionally, most of the research so far has been directed towards identifying the inhibitors that are selective for FAP over dipeptidyl peptidases (DPPs), with which it shares exopeptidase specificity, and over cytosolic prolyl endopeptidase (PREP), with which it shares endopeptidase specificity [16].

Interestingly, FAP has been reported to exhibit an absolute requirement for glycine at the P2 position as an endopeptidase [17,18]. This may explain why most small-molecule FAP inhibitors are based upon an X-Gly-Pro sequence. Jansen et al. presented *N*-(4-quinolinoyl)-Gly-(2-cyanopyrrolidine) as an extremely potent FAP inhibitor (IC_50_ = 10.3 ± 0.4 nM), with high selectivity indices (SI) over both DPPs (>10^3^) and PREP (>83-fold) [19]. Remarkably, the substitution of the nitrile on the P1 pyrrolidine ring with a more electrophilic boronic acid warhead yielded *N*-(4-quinolinoyl)-Gly-boroPro that demonstrated 2.8-fold higher FAP binding affinity (IC_50_ = 3.7 ± 0.2 nM) than the former. Moreover, it retained selectivity over DPPs (>10^3^) with a ~3-fold selectivity over PREP [14].

Contrary to the notion that FAP exhibits an absolute requirement for glycine at the P2 position, two different groups identified small-molecule FAP inhibitors bearing a P2 D-alanine. Tran et al. were the first to show FAP’s tolerability for a P2 d-alanine [20]. They reported *N*-acetyl-D-Ala-boroPro to exhibit a modest potency with a K_i_(FAP) of 350 nM, compared to *N*-acetyl-Gly-boroPro that possessed a K_i_(FAP) of 23 nM. In the study published by Bachovchin and co-workers, *N*-acetyl-D-Ala-boroPro was also shown to have a modest FAP binding potency (IC_50_ = 2900 ± 600 nM) [21]. Importantly, in the same study, *N*-(pyridine-4-carbonyl)-D-Ala-boroPro (ARI-3099) was identified as a highly potent FAP inhibitor with an IC_50_ of 36 ± 4.8 nM. Its P2 glycine congener was reported to have a ~75-fold higher FAP binding affinity. Notably, compared to *N*-(benzoyl)-D-Ala-boroPro (IC_50_(FAP) = 54 ± 2.9 nM), *N*-(4-quinolinoyl)-D-Ala-boroPro (IC_50_(FAP) = 6.4 ± 1.4 nM) performed superiorly with ~8-fold higher FAP binding affinity. The FAP/PREP selectivity values were 160 and 33 for the quinoline- and benzoyl-based inhibitors, respectively, indicating that the quinoline-based inhibitors are more selective for binding to FAP. Nevertheless, the findings from the two studies validated FAP’s ability to tolerate a P2 D-alanine in the context of an X-boroPro-based inhibitor.

**Figure 1:**
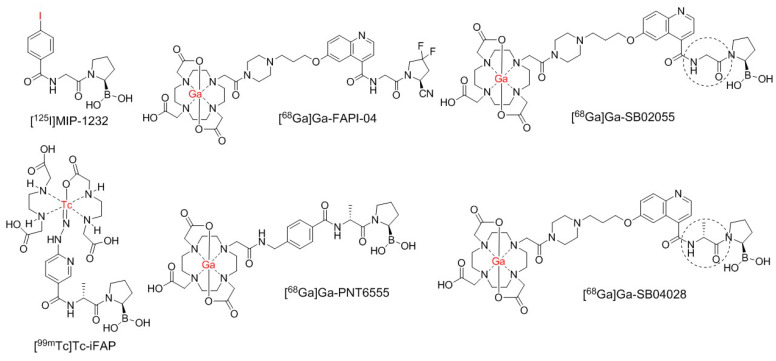
Chemical structures of FAP-targeted ligands [^68^Ga]Ga-SB02055 and [^68^Ga]Ga-SB04028, and previously reported [^125^I]MIP-1232, [^99m^Tc]Tc-iFAP, [^68^Ga]Ga-FAPI-04 and [^68^Ga]Ga-PNT6555. The difference between [^68^Ga]Ga-SB02055 and [^68^Ga]Ga-SB04028 is indicated by dotted circles. The radioisotopes are in red.

The radiolabeled small-molecule FAP inhibitor called [^125^I]MIP-1232 (Figure 1) was reported in 2015 and was based on (*R*)-(1-(benzoylglycyl)pyrrolidin-2-yl)boronic acid framework [22]. Although the probe did not serve its primary purpose of atherosclerosis imaging, the authors presented its relevance as a radiotracer for imaging FAP-positive tumor tissues. [^125^I]MIP-1232 was reported to strongly accumulate in FAP-positive SK-Mel-187 melanoma xenograft in vitro.

Recently, a new SPECT tracer [^99m^Tc]Tc-HYNIC-D-Ala-boroPro ([^99m^Tc]Tc-iFAP, Figure 1) targeting FAP was reported to exhibit 7.05 ± 1.13 %ID/g tumor uptake and 11.18 ± 1.54 %ID/g kidney uptake in an induced Hep-G2 tumor model in mice at 30 min post injection (pi) [23]. The tumor and kidney uptake values reportedly dropped by ~27% (5.18 ± 0.82 %ID/g) and ~33% (7.46 ± 1.02 %ID/g), respectively, at 2 h pi with minimal uptake in most normal organs. In its clinical study, [^99m^Tc]Tc-iFAP was seen to accumulate in the primary tumors and lymph node metastases of patients with different cancers [24]. The mean radiation equivalent dose for the kidney (5.2 ± 0.8 mSv) was stated to be more than twice the effective dose (2.3 ± 0.4 mSv) after administrating 740 MBq of the tracer. The authors concluded the study by declaring the need for additional clinical studies to further validate the performance of [^99m^Tc]Tc-iFAP.

The development of targeted radiotracers by Haberkorn group is one of the most significant and clinically relevant applications of FAP inhibitors to date. By expanding on the framework previously outlined by Jansen et al., they generated a panel of specific and high-affinity FAP inhibitors (FAPIs), undergoing almost complete internalization of the ligand-enzyme complex [25,26,27]. These derivatives were synthesized using different chemical modifications to attach various chelators via a linker to the quinolinoyl moiety in *N*-(4-quinolinoyl)-Gly-(2-cyanopyrrolidine). Of these, ^68^Ga-labeled FAPI-02 [28], FAPI-04 (Figure 1) and FAPI-46 [29], ^99m^Tc-labeled FAPI-34 [30] and ^18^F-labeled FAPI-42 [31] and FAPI-74 [32] are the most advanced and validated in clinical studies for cancer imaging.

Most recently, [^68^Ga]Ga/[^177^Lu]Lu/[^225^Ac]Ac-PNT6555 were presented as highly potent FAP-targeted theranostic pairs in two different conference abstracts [33,34]. PNT6555 (Figure 1) is a DOTA-conjugated FAP-targeted ligand based on a (*R*)-(1-((4-(aminomethyl)benzoyl)-d-alanyl)pyrrolidin-2-yl)boronic acid scaffold. [^177^Lu]Lu-PNT6555 was reported to show little retention in normal tissues but a high level of tumor retention (>10 %ID/g) was observed up to 168 h pi. A single dose of [^177^Lu]Lu/[^225^Ac]Ac-PNT6555 was reported to exhibit a dose-dependent anti-tumor efficacy in a HEK-mFAP murine tumor model and a phase I clinical trial of [^177^Lu]Lu-PNT6555 is currently underway [35]. No peer-reviewed publications on imaging or ex vivo biodistribution analysis of [^68^Ga]Ga-, [^177^Lu]Lu- and/or [^225^Ac]Ac-PNT6555 are currently available.

There are varying preclinical and clinical reports on the association between the uptake intensity of radiolabeled FAPIs and FAP expression. For example, while [^68^Ga]Ga-FAPI-04 is observed to have a high uptake in the bone and muscle in mice, the former is not observed in humans [26,36,37,38,39,40]. However, the non-tumor-specific uptake of [^68^Ga]Ga-FAPI-04 and [^68^Ga]Ga-FAPI-46 was recently reported in muscles and degenerative lesions mostly associated with the joints and vertebral bones [39]. Although having additional clinical studies would help ascertain these ambiguous associations, efforts towards the development of FAP-targeted inhibitors with improved pharmacokinetic properties and enhanced FAP specificity compared to the already existing FAPIs is warranted.

Compared to nitrile, the boronic acid warhead has been reported to have a higher FAP binding affinity [14,19], possibly due to its stronger electrophilic character, and it could have a faster pharmacokinetics in vivo due to its highly hydrophilic nature. Furthermore, previous findings confirm that FAP can tolerate a P2 D-alanine apart from a P2 glycine [20,21]. Lastly, a P3 quinolinoyl moiety has been previously optimized for developing radioligands for FAP targeting by Haberkorn et al. [25,27]. With this aim in mind, we designed two novel boronic acid-based DOTA-conjugated FAP-targeted ligands; [^68^Ga]Ga-SB02055 and [^68^Ga]Ga-SB04028 (Figure 1), bearing a *N*-(4-quinolinoyl)-Gly-boroPro and *N*-(4-quinolinoyl)-d-Ala-boroPro pharmacophores, respectively. The addition of chelator DOTA via a piperazine-based linker (Figure 1) allowed the formation of stable complexes with the PET isotope ^68^Ga. We posited that these novel boronic acid-based tracers may exhibit a higher tumor uptake by virtue of their more potent FAP inhibition and demonstrate superior pharmacokinetics by undergoing quick clearance from all the non-target organs/tissues and serve as PET imaging agents with excellent imaging contrast. Both tracers were subjected to a thorough preclinical evaluation including in vitro FAP binding assays, in vivo stability testing and, PET/CT imaging and ex vivo biodistribution studies using HEK293T:hFAP tumor xenograft mouse model. The results were then compared with those obtained from [^68^Ga]Ga-PNT6555, which is currently been evaluated in the clinic [35].

## 2. Results

### 2.1. Synthesis of ^68^Ga- and ^nat^Ga-Complexed DOTA-Conjugated FAP-Targeted Ligands

**Scheme 1:**
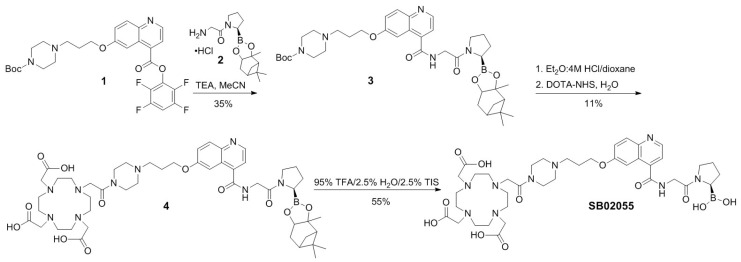
Synthesis of DOTA-conjugated precursor SB02055.

Detailed information on the synthesis, purification and characterizations of all intermediates, precursors and ^nat^Ga/^68^Ga-complexed ligands is provided in the Supplementary Materials. The identities of intermediates, precursors and nonradioactive Ga-complexed standards were confirmed by MS and/or NMR analyses (Figures S1–S11). For the synthesis of SB02055 (Scheme 1), compound **1** [41] was coupled overnight with compound **2** [20,42] to obtain compound **3** in a 35% yield. Compound **3** was Boc-deprotected by stirring it overnight with diethyl ether (Et₂O):4M HCl/dioxane (*v*/*v*) in a 1:1 ratio. The mixture was then evaporated before reacting it with DOTA-NHS to afford compound **4** as a pinanediol ester of precursor SB02055 in 11% yield over two steps. Compound **4** was treated with a cleavage cocktail consisting of 95% trifluoroacetic acid (TFA)/2.5% H_2_O/2.5% triisopropylsilane (TIS) for 4 h at room temperature (RT) to finally afford the desired precursor SB02055 in 55% yield.

**Scheme 2:**
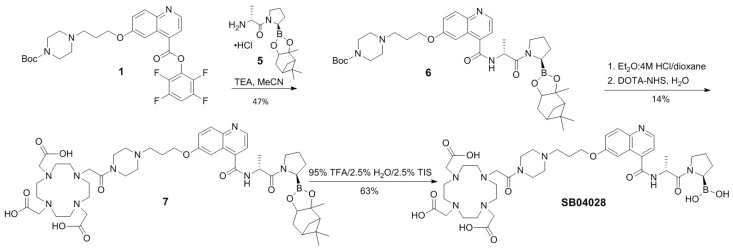
Synthesis of DOTA-conjugated precursor SB04028.

For the synthesis of SB04028 (Scheme 2), compound **5** prepared following the literature procedures [23] was coupled with compound **1** overnight to afford compound **6** in a 47% yield. Compound **6** was then treated overnight with Et_2_O:4M HCl/dioxane (*v*/*v*) in a 1:1 ratio for removing the Boc-group. The resulting mixture was evaporated before reacting it with DOTA-NHS to obtain compound **7** as a pinanediol ester of SB04028 in 14% yield over two steps. Finally, compound **7** was treated with a cleavage cocktail consisting of 95% TFA/2.5% H_2_O/2.5% TIS for 4 h at RT to yield the desired precursor SB04028 in a 63% yield.

^nat^Ga-complexed standards were prepared by incubating the respective precursors with excess ^nat^GaCl_3_ in NaOAc buffer (0.1 N, pH 4.5), followed by HPLC purification [43]. ^nat^Ga-SB02055, ^nat^Ga-SB04028 and ^nat^Ga-PNT6555 were obtained in 36–98% yields. ^68^Ga labeling was performed in HEPES buffer (2 M, pH 5.0) [43]. HPLC purification of the crude reaction yielded the desired ^68^Ga-labeled analogues in 19–58% decay-corrected radiochemical yield with >92% radiochemical purity and >9.1 GBq/µmol molar activity.

### 2.2. In Vitro Fluorescence-Based Binding Assay

**Figure 2:**
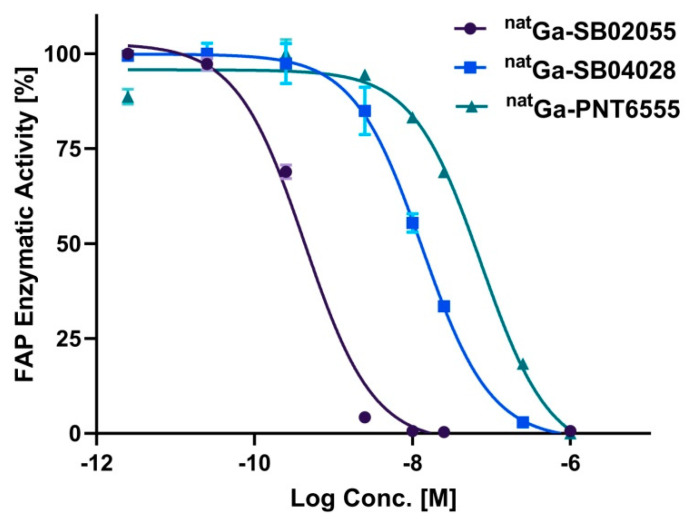
Inhibition of rhFAP-mediated cleavage of a fluorogenic substrate (Suc-Gly-Pro-AMC) by varying concentrations of ^nat^Ga-SB02055, ^nat^Ga-SB04028 and ^nat^Ga-PNT6555.

The enzymatic cleavage of a fluorogenic substrate (Suc-Gly-Pro-AMC) by the recombinant human FAP (rhFAP) was inhibited by ^nat^Ga-SB02055, ^nat^Ga-SB04028 and ^nat^Ga-PNT6555 in a dose-dependent manner (Figure 2). IC_50_ values calculated for ^nat^Ga-SB02055, ^nat^Ga-SB04028 and ^nat^Ga-PNT6555 were found to be 0.41 ± 0.06, 13.9 ± 1.29 and 78.1 ± 4.59 nM, respectively (*n* = 3).

### 2.3. Ex Vivo Biodistribution and PET/CT Imaging Studies

**Figure 3:**
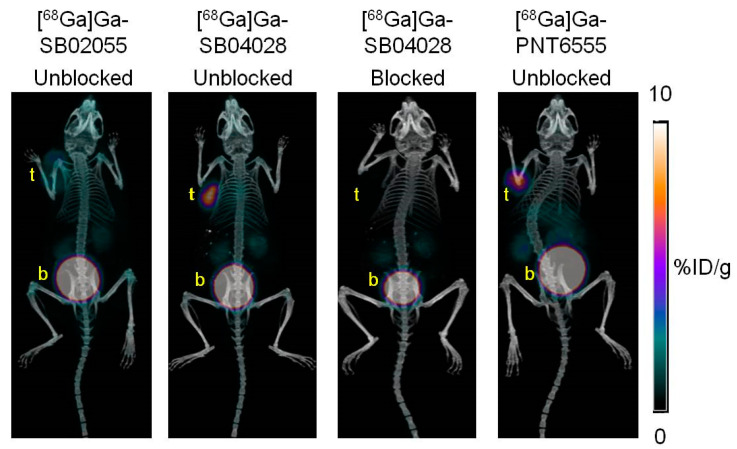
Representative maximum intensity projection PET/CT images of [^68^Ga]Ga-SB02055 (unblocked), [^68^Ga]Ga-SB04028 (unblocked and blocked) and [^68^Ga]Ga-PNT6555 (unblocked) at 1 h pi in HEK293T:hFAP tumor-bearing male NRG mice. The mice in the blocked group were co-injected with FAPI-04 (0.5 mg/mouse). t: tumor; b: urinary bladder.

The PET/CT image acquired at 1 h pi with [^68^Ga]Ga-SB04028 showed a clear visualization of the HEK293T:hFAP tumor xenograft [44], with the tumor uptake being comparatively higher than [^68^Ga]Ga-PNT6555 (Figure 3). Moreover, [^68^Ga]Ga-SB04028 displayed a faster clearance from most normal organs/tissues, with a relatively lower kidney uptake and slightly superior tumor/background contrast ratios compared to [^68^Ga]Ga-PNT6555. On the other hand, while the uptake of [^68^Ga]Ga-SB02055 in most normal organs/tissues was minimal, the tumor uptake of [^68^Ga]Ga-SB02055 was also negligible on the PET/CT image (Figure 3). A high accumulation of all the three radiotracers in the urinary bladder indicated excretion via the renal pathway. The co-injection of [^68^Ga]Ga-SB04028 with FAPI-04 (0.5 mg/mouse) reduced the tumor uptake to the background level (Figure 3).

**Figure 4:**
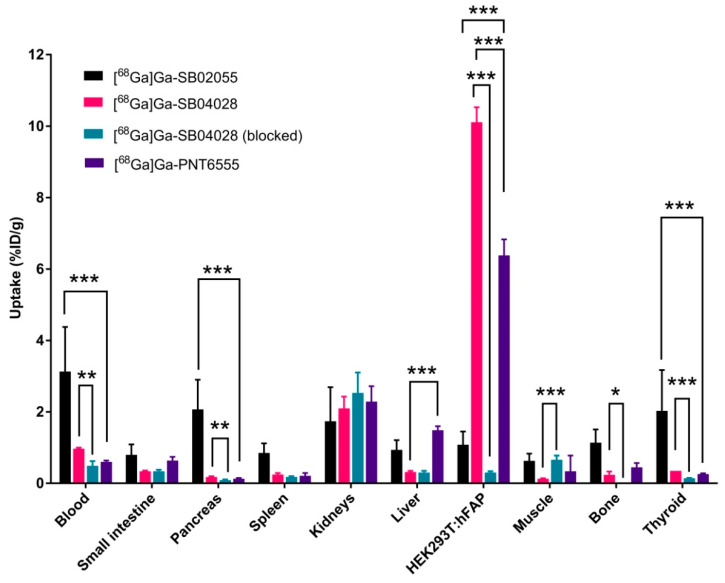
Ex vivo biodistribution data of [^68^Ga]Ga-SB02055, [^68^Ga]Ga-SB04028 and [^68^Ga]Ga-PNT6555 in HEK293T:hFAP tumor-bearing male NRG mice at 1 h pi (*n* = 4). The mice in the blocked group were co-injected with FAPI-04 (0.5 mg/mouse). * *p* < 0.05; ** *p* < 0.01, *** *p* < 0.001.

Data from biodistribution studies (Figure 4 and Table S1) for all three tracers conducted at 1 h pi in HEK293T:hFAP tumor-bearing mice were found to be concordant with their respective PET/CT images. The tumor uptake values were found to be 1.08 ± 0.37, 10.1 ± 0.42 and 6.38 ± 0.45 %ID/g for [^68^Ga]Ga-SB02055, [^68^Ga]Ga-SB04028 and [^68^Ga]Ga-PNT6555, respectively. All the tracers were excreted rapidly via the renal pathway with a low but comparable kidney uptake (1.74 ± 0.95 %ID/g for [^68^Ga]Ga-SB02055, 2.10 ± 0.33 %ID/g for [^68^Ga]Ga-SB04028 and 2.29 ± 0.43 %ID/g for [^68^Ga]Ga-PNT6555). [^68^Ga]Ga-SB02055 displayed a retention slighter higher than the background muscle uptake (0.63 ± 0.20 %ID/g) in some normal organs, particularly the blood (3.13 ± 1.25 %ID/g), pancreas (2.07 ± 0.83 %ID/g), bone (1.14 ± 0.37 %ID/g) and thyroid (1.49 ± 0.47 %ID/g). Akin to the control tracer [^68^Ga]Ga-PNT6555, [^68^Ga]Ga-SB04028 exhibited a minimal uptake in most normal organs/tissues, but demonstrated superior tumor/background contrast ratios than [^68^Ga]Ga-PNT6555.

**Figure 5:**
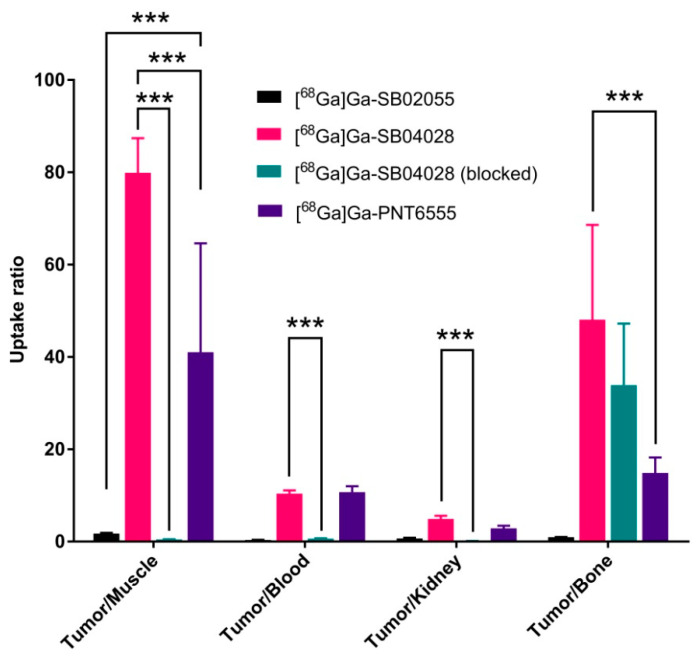
Tumor/organ uptake ratios of [^68^Ga]Ga-SB02055, [^68^Ga]Ga-SB04028 and [^68^Ga]Ga-PNT6555 in HEK293T:hFAP tumor-bearing male NRG mice at 1 h pi (*n* = 4). The mice in the blocked group were co-injected with FAPI-04 (0.5 mg/mouse). *** *p* < 0.001.

The tumor/muscle, tumor/kidney and tumor/bone ratios for [^68^Ga]Ga-SB04028 were considerably higher than those for [^68^Ga]Ga-PNT6555 (79.9 ± 7.50 vs. 52.6 ± 5.86, 4.89 ± 0.72 vs. 2.87 ± 0.59 and 48.1 ± 20.5 vs. 14.9 ± 3.34, respectively) (Figure 5 and Table S1). These uptake ratios were significantly lower for [^68^Ga]Ga-SB02055 (tumor/muscle: 1.72 ± 0.19; tumor/kidney: 0.67 ± 0.15; tumor/bone: 0.95 ± 0.06) compared to both control [^68^Ga]Ga-PNT6555 as well as [^68^Ga]Ga-SB04028. Additionally, the tumor/blood ratios for [^68^Ga]Ga-SB04028 (10.4 ± 0.70) and [^68^Ga]Ga-PNT6555 (10.7 ± 1.29) were comparable and the tumor/blood ratio was much lower for [^68^Ga]Ga-SB02055 (0.35 ± 0.03).

The co-injection of [^68^Ga]Ga-SB04028 with FAPI-04 (0.5 mg/mouse) reduced the average tumor uptake by ~97% (10.1 ± 0.42 %ID/g vs. 0.30 ± 0.04 %ID/g) (Figure 4 and Table S1). No significant reduction was observed in its kidney uptake (2.10 ± 0.33 %ID/g vs. 2.53 ± 0.57 %ID/g) upon FAPI-04 co-injection.

### 2.4. In Vivo Stability Studies

The in vivo stability studies revealed [^68^Ga]Ga-SB04028 to be considerably stable in the blood plasma of male NRG mice (*n* = 3) at 15 min pi compared to [^68^Ga]Ga-SB02055. While no intact [^68^Ga]Ga-SB02055 was present in the mouse plasma at 15 min pi, 46.6 ± 0.21% of radioactivity from [^68^Ga]Ga-SB04028 was found to be present as an intact tracer. The fractions of tracers remaining intact in the urine at 15 min pi for [^68^Ga]Ga-SB02055 and [^68^Ga]Ga-SB04028 were 93.5 ± 0.91% and 53.6 ± 7.11%, respectively (Figures S1–S4).

## 3. Discussion

The FAP binding affinity of ^nat^Ga-SB02055 (0.41 ± 0.06 nM) was found to be ~190-fold higher than ^nat^Ga-PNT6555 (78.1 ± 4.59 nM), whereas that of ^nat^Ga-SB04028 (13.9 ± 1.29 nM) was ~5.6-fold higher than ^nat^Ga-PNT6555 in the rhFAP enzymatic assays (Figure 2). This is consistent with previous reports demonstrating bicyclic heteroaromatic quinolinoyl-based FAP inhibitors to be more potent than their monocyclic homoaromatic benzoyl-based congeners [13,14,19,21,45]. Additionally, FAP seems to prefer P2 glycine (SB02055) over a P2 d-alanine (SB04028). It is to be noted that P2 glycine bearing *N*-(4-quinolinoyl)-Gly-boroPro was observed to exhibit an IC_50_(FAP) of 3.7 ± 0.2 nM by Jansen and co-workers [14]. Interestingly, its P2 D-alanine analogue, *N*-(4-quinolinoyl)-d-Ala-boroPro, was shown to exhibit an IC_50_(FAP) of 6.4 ± 1.4 nM only by Bachovchin et al. [21] in a separate study, and hence cannot be pitted head to head for comparison.

Surprisingly, findings from the in vitro enzymatic assay for ^nat^Ga-SB02055 were not in concordance with the data from the PET/CT imaging and biodistribution analysis of its radioactive counterpart obtained from HEK293T:hFAP tumor-bearing mice at 1 h pi. [^68^Ga]Ga-SB02055 exhibited a nominal tumor uptake (1.08 ± 0.37 %ID/g) close to the background levels that was ~6-fold lower than [^68^Ga]Ga-PNT6555 (6.38 ± 0.45 %ID/g) and primarily underwent renal excretion (Figure 3 and Figure 4, Table S1). However, the uptake in normal organs, particularly the blood (3.13 ± 1.25 %ID/g), pancreas (2.07 ± 0.83 %ID/g), bone (1.14 ± 0.37 %ID/g) and thyroid (1.49 ± 0.47 %ID/g), was greater than the background muscle uptake (0.63 ± 0.20 %ID/g).

We initially suspected the lack of in vivo stability of [^68^Ga]Ga-SB02055 to be a reason for its nominal tumor uptake. Upon subjecting it to in vivo stability testing at 15 min pi, we found no intact [^68^Ga]Ga-SB02055 in the mouse plasma. Interestingly, the urine samples collected at the same time presented with >93% intact [^68^Ga]Ga-SB02055 (Figures S1 and S2). The low tumor uptake at 1 h pi could possibly be a consequence of [^68^Ga]Ga-SB02055 undergoing quick clearance from the blood and rapid renal excretion into the urine in ≤15 min of injecting the tracer in vivo. Furthermore, an unidentified highly hydrophilic ^68^Ga-labeled species accounting for >99% plasma fraction was observed at 15 min pi during the stability testing. The higher than background level uptake in the blood could supposedly be attributed to this uncharacterized ^68^Ga-labeled species. Further investigation of [^68^Ga]Ga-SB02055 in this regard is currently underway.

Next, encouraged by earlier findings regarding FAP’s ability to tolerate a P2 D-alanine in the context of an X-boroPro-based inhibitor [20,21], we sought to evaluate the effect of substituting the P2 glycine in [^68^Ga]Ga-SB02055 with a D-alanine to yield [^68^Ga]Ga-SB04028. [^68^Ga]Ga-SB04028 differed from [^68^Ga]Ga-SB02055 in only the P2 amino acid residue, while rest of the molecular structure was kept alike.

The PET/CT imaging data were consistent with the data from the biodistribution analysis performed at 1 h pi in HEK293T:hFAP tumor-bearing mice. [^68^Ga]Ga-SB04028 demonstrated >1.5-fold higher tumor uptake (10.1 ± 0.42 %ID/g) than [^68^Ga]Ga-PNT6555 (6.38 ± 0.45 %ID/g) (Figure 3 and Figure 4, Table S1) and concordant with the findings from FAP inhibition assays where ^nat^Ga-SB04028 was realized to be more potent than ^nat^Ga-PNT6555. Additionally, the high accumulation of both [^68^Ga]Ga-SB04028 and [^68^Ga]Ga-PNT6555 in the bladder indicated their renal excretion. The uptake of [^68^Ga]Ga-SB04028 in most normal organs/tissues was minimal and comparable to [^68^Ga]Ga-PNT6555, particularly in the muscle (0.13 ± 0.01 vs. 0.12 ± 0.02 %ID/g), blood (0.97 ± 0.03 vs. 0.60 ± 0.04 %ID/g), kidney (2.10 ± 0.33 vs. 2.29 ± 0.43 %ID/g) and bone (0.24 ± 0.09 vs. 0.45 ± 0.12 %ID/g) (Figure 4 and Table S1). However, since the tumor uptake of [^68^Ga]Ga-SB04028 was considerably greater than [^68^Ga]Ga-PNT6555, the corresponding tumor/organ uptake ratios for [^68^Ga]Ga-SB04028 were found to be significantly higher than [^68^Ga]Ga-PNT6555 (tumor/muscle: 79.9 ± 7.50 vs. 52.6 ± 5.86; tumor/kidney: 4.89 ± 0.72 vs. 2.87 ± 0.59; tumor/bone: 48.1 ± 20.5 vs. 14.9 ± 3.34), except tumor/blood ratios (10.4 ± 0.70 vs. 10.7 ± 1.29 %ID/g), which were equivalent for the two (Figure 5 and Table S1). In general, [^68^Ga]Ga-SB04028 demonstrated superior tumor/background contrast ratios compared to [^68^Ga]Ga-PNT6555 as evident from both PET/CT imaging (Figure 3) and biodistribution analysis (Figure 5 and Table S1).

The imaging and biodistribution analysis of mice co-injected [^68^Ga]Ga-SB04028 with FAPI-04 (0.5 mg/mouse) revealed a ~97% reduction in the tumor uptake (10.1 ± 0.42 %ID/g vs. 0.30 ± 0.04 %ID/g) substantiating in vivo FAP specificity of our lead candidate [^68^Ga]Ga-SB04028 (Figure 3 and Figure 4, Table S1). Moreover, as anticipated since there was no significant reduction observed in the kidney uptake of [^68^Ga]Ga-SB04028 (2.10 ± 0.33 %ID/g vs. 2.53 ± 0.57 %ID/g), we can conclusively state that retention in the kidneys is by virtue of the tracer’s propensity to undergo renal elimination and is not FAP mediated.

Unlike its P2 glycine analogue [^68^Ga]Ga-SB02055, [^68^Ga]Ga-SB04028 with a P2 D-alanine residue was found to be relatively more stable during in vivo stability testing. Strikingly, >47% of [^68^Ga]Ga-SB04028 remained intact in the mouse plasma at 15 min pi (Figures S3 and S4). The remaining fractions corresponded to currently uncharacterized but highly hydrophilic ^68^Ga-labeled species. The urine samples from the mouse presented with >54% intact tracer, with the remaining portions co-eluting more or less with the uncharacterized ^68^Ga-labeled species found in the plasma samples for [^68^Ga]Ga-SB04028.

The exact nature and identity of the ^68^Ga-labeled species eluting prior to each of the intact tracers during in vivo stability testing has not been characterized. However, it has been shown that boronic acids under basic (physiological) conditions could be converted to other species. These include: (1) the degradation products formed under physiological pH conditions due to the inherent susceptibility of boronic acids to undergo oxidation [46,47]; the empty p orbital of boron in boronic acid is prone to attack by nucleophilic oxygen of reactive oxygen species (ROS), resulting in the formation of labile boric esters and/or oxidative de-boronation products; notably, it is the same attribute that allows boronic acid-based ligands to react with the nucleophilic hydroxyl of the catalytic serine residue of FAP and form reversible covalent adducts; (2) the anionic boronic acid/hydroxyboronate anion formed under more basic conditions as a result of hydroxyl (OH^−^) attack on the highly electrophilic boron atom [48]; naturally, these ionic species will have a tendency to elute before the neutral boronic acid forms; and (3) boronate esters formed under aqueous conditions at a physiological pH of ~7.4 [48]. Efforts are underway to characterize these species.

The goal of the present work was to develop novel boroPro-based PET imaging agents with superior pharmacokinetics properties and excellent imaging contrast for easy and reliable diagnosis of FAP-overexpressing carcinomas. Although our lead candidate [^68^Ga]Ga-SB04028 demonstrated superior tumor/background contrast ratios compared to [^68^Ga]Ga-PNT6555, there is still some scope for further optimization. (1) A 4,4-difluoro substituent at the (2*S*)-2-cyanopyrrolidine ring was previously reported to enhance FAP inhibitory activity compared to that of unsubstituted analogue [12,13,14]. It would be interesting to generate a difluorinated derivative of [^68^Ga]Ga-SB04028 for preclinical investigation, although its synthesis might be a bit challenging. (2) In terms of diagnostic radiopharmaceuticals, ^99m^Tc is the most widely used with SPECT imaging accounting for >70% of all the imaging procedures performed in the field of nuclear medicine. Replacing DOTA with a suitable chelator to allow radiolabeling with ^99m^Tc is definitely worth exploiting.

## 4. Materials and Methods

### 4.1. Synthesis of ^nat^Ga and ^68^Ga-Complexed DOTA-Conjugated FAP-Targeted Ligands

Detailed information on the synthesis, purification and characterizations of the intermediates, precursors (SB02055 and SB04028), ^nat^Ga-complexed analogues (^nat^Ga-SB02055, ^nat^Ga-SB04028 and ^nat^Ga-PNT6555) and their ^68^Ga-complexed analogues ([^68^Ga]Ga-SB02055, [^68^Ga]Ga-SB04028 and [^68^Ga]Ga-PNT6555) is provided in the Supplementary Materials.

### 4.2. Cell Culture

HEK293T cells were obtained from American Type Culture Collection (Manassas, VA, USA). The IMPACT Rodent Pathogen Test (IDEXX BioAnalytics, Columbia, MO, USA) confirmed that the cells were pathogen free. Detailed information on the generation of HEK293T:hFAP cells has been previously reported by our group [44]. HEK293T:hFAP cells were cultured in DMEM GlutaMAX™ medium supplemented with 10% FBS, penicillin (100 U/mL) and streptomycin (100 µg/mL) at 37 °C in a Panasonic Healthcare (Tokyo, Japan) MCO-19AIC humidified incubator containing 5% CO_2_. Cells were grown until 80–90% confluence, washed with sterile PBS (pH 7.4) and collected.

### 4.3. In Vitro Fluorescence-Based Binding Assay

The half maximal inhibitory concentration (IC_50_) values of the FAP-targeted ligands were measured using in vitro enzymatic assay following previously reported procedures [41,44]. Briefly, rhFAP (Biolegend, San Diego, CA, USA; 0.2 µg/mL, 50 µL) was added into a Costar clear bottom 96-well plate. Varied concentrations (25 pM to 1 µM) of ^nat^Ga-complexed ligands were added to each well (in duplicate) containing the rhFAP. PBS was added to the control well (in duplicate). After incubating for 30 min at 37 °C, the fluorescence substrate Suc-Gly-Pro-AMC (Bachem, Bubendorf, Switzerland; 20 mM, 50 µL) was added to each well. The velocities of AMC release were measured kinetically at λ_ex_ = 380 nm, λ_em_ = 460 nm at 60 min at 37 °C using a FlexStation 3 Multi-Mode Microplate Reader.

### 4.4. Ex Vivo Biodistribution and PET/CT Imaging Studies

Imaging and biodistribution studies were performed using immunodeficient male NRG (NOD.Cg-Rag1tm1Mom Il2rgtm1Wjl/SzJ) mice following previously reported procedures [41,44]. All experiments were conducted according to the guidelines established by the Canadian Council on Animal Care and approved by Animal Ethics Committee of the University of British Columbia. The mice were subcutaneously inoculated with 7.5 × 10^6^ HEK293T:hFAP cells in the left dorsal flank. When the tumors grew to 6–8 mm in diameter, the mice were used for PET/CT imaging and biodistribution studies.

The PET/CT imaging experiments were carried out using a Siemens Inveon micro PET/CT scanner (Knoxville, TN, USA). Tumor-bearing mice were injected with ~4 to 6 MBq of ^68^Ga-labeled tracer through a lateral caudal tail vein under 2.5% isoflurane in oxygen anesthesia, followed by recovery and free roaming in their cage during the uptake period. A 10 min localization CT scan was acquired using 3 overlapping positions to cover the entire mouse. The CT scan was used for the attenuation and scatter correction, and anatomical localization. A list mode acquisition was then performed for 15 min at 1 h pi with the mouse under isoflurane sedation. The images were reconstructed using 3D OSEM/MAP iterative methods.

For the biodistribution studies, the mice were injected with ~1–2 MBq of the ^68^Ga-labeled tracer, using the exact procedures as described above. At 1 h pi, the mice were euthanized by CO_2_ inhalation. Blood was withdrawn by a cardiac puncture, and the organs/tissues of interest were collected, weighed and counted using a Perkin Elmer (Waltham, MA, USA) Wizard2 2480 automatic gamma counter. The uptake values in percent of injected dose per gram of tissues (%ID/g) for the different tracers were calculated.

For blocking studies, HEK293T:hFAP tumor-bearing mice were co-injected [^68^Ga]Ga-SB04028 with an excess of FAPI-04 (0.5 mg/mouse) as a competitor. Imaging and biodistribution studies were performed at 1 h pi similar to the unblocked mice.

### 4.5. In Vivo Stability Studies

In vivo stability studies were performed using healthy (non-tumor-bearing) male NRG mice following previously reported procedures [43,49]. Briefly, [^68^Ga]Ga-SB02055 (6.32 ± 0.16 MBq) and [^68^Ga]Ga-SB04028 (9.90 ± 0.26 MBq) were injected via the lateral caudal vein into the mice (*n* = 3). At 15 min pi, the mice were sedated and euthanized, and urine and blood were collected. The blood was processed to obtain plasma by adding 0.5 mL of acetonitrile to 0.5–0.8 mL of whole blood, then vortexing and centrifuging the resulting mixture on a benchtop mini-centrifuge (Fisherbrand™ Mini-Centrifuge 100–240 V, 50/60 Hz Universal Plug) for 20 min. The supernatant was collected and filtered to give the blood plasma. The plasma and urine samples were analyzed via radio-HPLC by using the same conditions as those used for quality control of these ^68^Ga-labeled radioligands.

### 4.6. Statistical Analysis

Data were reported as mean ± standard deviation (SD) and analyzed with GraphPad Prism, version 9.5.1. Two-way ANOVA and multiple *t*-tests were performed for all the tumor and organ/tissue uptake values in the biodistribution studies of [^68^Ga]Ga-SB02055, [^68^Ga]Ga-SB04028, and [^68^Ga]Ga-PNT6555 in the HEK293T:hFAP tumor model. Statistical significance was defined at *p* < 0.05 using the Holm–Sidak method.

## 5. Conclusions

We successfully synthesized and evaluated two novel ^68^Ga-labeled (*R*)-pyrrolidin-2-yl-boronic acid-derived PET tracers for FAP-targeted cancer imaging. While the P2 glycine-containing [^68^Ga]Ga-SB02055 displayed a minimal tumor uptake, its P2 D-alanine congener [^68^Ga]Ga-SB04028 demonstrated a significantly higher tumor uptake and superior imaging contrast compared to [^68^Ga]Ga-PNT6555. (*R*)-(((quinoline-4-carbonyl)-D-alanyl)pyrrolidin-2-yl)boronic acid is a highly promising pharmacophore that can be used to develop PET imaging agents with superior pharmacokinetics properties and excellent imaging contrasts for visualization of FAP-overexpressing carcinomas. The replacement of DOTA with suitable chelators/prosthetic groups to allow radiolabeling with two of the most widely used imaging isotopes, ^99m^Tc and ^18^F, is warranted in the future.

## Data Availability

Data is contained within the article and Supplementary Materials.

## Supplementary Materials

The following supporting information can be downloaded at: https://www.mdpi.com/article/10.3390/ph16060798/s1. Detailed synthetic procedures and results for the preparation of FAP-targeted ligands and their ^nat^Ga/^68^Ga-complexed analogues; Table S1: Biodistribution and tumor/organ uptake ratios of [^68^Ga]Ga-SB02055, [^68^Ga]Ga-SB04028 and [^68^Ga]Ga-PNT6555 in HEK239T:hFAP tumor-bearing mice; Figure S1: A representative MS spectrum of compound **3**; Figure S2: A representative NMR spectrum of compound **3**; Figure S3: A representative MS spectrum of compound **4**; Figure S4: A representative MS spectrum of SB02055; Figure S5: A representative MS spectrum of ^nat^Ga-SB02055; Figure S6: A representative MS spectrum of compound **6**; Figure S7: A representative NMR spectrum of compound **6**; Figure S8: A representative MS spectrum of compound **7**; Figure S9: A representative MS spectrum of SB04028; Figure S10: A representative MS spectrum of ^nat^Ga-SB04028; Figure S11: A representative MS spectrum of ^nat^Ga-PNT6555; Figure S12: Representative radio-HPLC chromatograms from an in vivo stability study performed to determine the percent of the intact fraction of [^68^Ga]Ga-SB02055 in mouse plasma and urine samples collected at 15 min post-injection; Figure S13: Radio-HPLC analysis of [^68^Ga]Ga-SB02055; Figure S14: Representative radio-HPLC chromatograms from an in vivo stability study performed to determine the percent of the intact fraction of [^68^Ga]Ga-SB04028 in mouse plasma and urine samples collected at 15 min post-injection; Figure S15: Radio-HPLC analysis of [^68^Ga]Ga-SB04028.

## References

[1] Xing F., Saidou J., Watabe K. (2010). Cancer Associated Fibroblasts (CAFs) in Tumor Microenvironment. Front. Biosci. Landmark Ed..

[2] Garin-Chesa P., Old L.J., Rettig W.J. (1990). Cell Surface Glycoprotein of Reactive Stromal Fibroblasts as a Potential Antibody Target in Human Epithelial Cancers. Proc. Natl. Acad. Sci. USA.

[3] Scanlan M.J., Raj B.K.M., Calvo B., Garin-Chesa P., Sanz-Moncasi M.P., Healey J.H., Old L.J., Rettig W.J. (1994). Molecular Cloning of Fibroblast Activation Protein alpha, a Member of the Serine Protease Family Selectively Expressed in Stromal Fibroblasts of Epithelial Cancers. Proc. Natl. Acad. Sci. USA.

[4] Tchou J., Zhang P.J., Bi Y., Satija C., Marjumdar R., Stephen T.L., Lo A., Chen H., Mies C., June C.H. (2013). Fibroblast Activation Protein Expression by Stromal Cells and Tumor-associated Macrophages in Human Breast Cancer. Hum. Pathol..

[5] Kesch C., Yirga L., Dendl K., Handke A., Darr C., Krafft U., Radtke J.P., Tschirdewahn S., Szarvas T., Fazli L. (2021). High Fibroblast-activation-protein Expression in Castration-resistant Prostate Cancer Supports the Use of FAPI-molecular Theranostics. Eur. J. Nucl. Med. Mol. Imaging.

[6] Cheng J.D., Valianou M., Canutescu A.A., Jaffe E.K., Lee H.-O., Wang H., Lai J.H., Bachovchin W.W., Weiner L.M. (2005). Abrogation of Fibroblast Activation Protein Enzymatic Activity Attenuates Tumor Growth. Mol. Cancer Ther..

[7] Scott A.M., Wiseman G., Adjei A., Lee F.-T., Hopkins W., Divgi C.R., Hanson L.H., Mitchell P., Gansen D.N., Larson S.M. (2003). A Phase I Dose-escalation Study of Sibrotuzumab in Patients with Advanced or Metastatic Fibroblast Activation Protein-positive cancer. Clin. Cancer Res..

[8] Welt S., Divgi C.R., Scott A.M., Garin-Chesa P., Finn R.D., Graham M., Carswell E.A., Cohen A., Larson S.M., Old L.J. (1994). Antibody Targeting in Metastatic Colon Cancer: A Phase I Study of Monoclonal Antibody F19 Against a Cell-surface Protein of Reactive Tumor Stromal Fibroblasts. J. Clin. Oncol..

[9] Riet T., Abken H. (2015). Chimeric Antigen Receptor T cells: Power Tools to Wipe Out Leukemia and Lymphoma. Expert Rev. Hematol..

[10] Sheykhhasan M., Manoochehri H., Dama P. (2022). Use of CAR T-cell for Acute Lymphoblastic Leukemia (ALL) Treatment: A review study. Cancer Gene Ther..

[11] Wen Y., Wang C.-T., Ma T.-T., Li Z.-Y., Zhou L.-N., Mu B., Leng F., Shi H.-S., Li Y.-O., Wei Y.-Q. (2010). Immunotherapy Targeting Fibroblast Activation Protein Inhibits Tumor Growth and Increases Survival in a Murine Colon Cancer Model. Cancer Sci..

[12] Tsai T.-Y., Yeh T.-K., Chen X., Hsu T., Jao Y.-C., Huang C.-H., Song J.-S., Huang Y.-C., Chien C.-H., Chiu J.-H. (2010). Substituted 4-Carboxymethylpyroglutamic Acid Diamides as Potent and Selective Inhibitors of Fibroblast Activation Protein. J. Med. Chem..

[13] Ryabtsova O., Jansen K., Van Goethem S., Joossens J., Cheng J.D., Lambeir A.-M., De Meester I., Augustyns K., Van der Veken P. (2012). Acylated Gly-(2-cyano)pyrrolidines as Inhibitors of Fibroblast Activation Protein (FAP) and the Issue of FAP/prolyl Oligopeptidase (PREP)-selectivity. Bioorganic Med. Chem. Lett..

[14] Jansen K., Heirbaut L., Verkerk R., Cheng J.D., Joossens J., Cos P., Maes L., Lambeir A.-M., De Meester I., Augustyns K. (2014). Extended Structure–Activity Relationship and Pharmacokinetic Investigation of (4-Quinolinoyl)glycyl-2-cyanopyrrolidine Inhibitors of Fibroblast Activation Protein (FAP). J. Med. Chem..

[15] Hu Y., Ma L., Wu M., Wong M.S., Li B., Corral S., Yu Z., Nomanbhoy T., Alemayehu S., Fuller S.R. (2005). Synthesis and Structure–activity Relationship of N-alkyl Gly-boro-Pro Inhibitors of DPP4, FAP, and DPP7. Bioorganic Med. Chem. Lett..

[16] Rosenblum J.S., Kozarich J.W. (2003). Prolyl peptidases: A Serine Protease Subfamily with High Potential for Drug Discovery. Curr. Opin. Chem. Biol..

[17] Edosada C.Y., Quan C., Wiesmann C., Tran T., Sutherlin D., Reynolds M., Elliott J.M., Raab H., Fairbrother W., Wolf B.B. (2006). Selective Inhibition of Fibroblast Activation Protein Protease Based on Dipeptide Substrate Specificity. J. Biol. Chem..

[18] Edosada C.Y., Quan C., Tran T., Pham V., Wiesmann C., Fairbrother W., Wolf B.B. (2006). Peptide Substrate Profiling Defines Fibroblast Activation Protein as an Endopeptidase of Strict Gly_2_-Pro_1_-cleaving Specificity. FEBS Lett..

[19] Jansen K., Heirbaut L., Cheng J.D., Joossens J., Ryabtsova O., Cos P., Maes L., Lambeir A.-M., De Meester I., Augustyns K. (2013). Selective Inhibitors of Fibroblast Activation Protein (FAP) with a (4-Quinolinoyl)-glycyl-2-cyanopyrrolidine Scaffold. ACS Med. Chem. Lett..

[20] Tran T., Quan C., Edosada C.Y., Mayeda M., Wiesmann C., Sutherlin D., Wolf B.B. (2007). Synthesis and Structure–activity Relationship of N-acyl-Gly-, N-acyl-Sar- and N-blocked-boroPro Inhibitors of FAP, DPP4, and POP. Bioorg. Med. Chem. Lett..

[21] Poplawski S.E., Lai J.H., Li Y., Jin Z., Liu Y., Wu W., Wu Y., Zhou Y., Sudmeier J.L., Sanford D.G. (2013). Identification of Selective and Potent Inhibitors of Fibroblast Activation Protein and Prolyl Oligopeptidase. J. Med. Chem..

[22] Meletta R., Herde A.M., Chiotellis A., Isa M., Rancic Z., Borel N., Ametamey S.M., Krämer S.D., Schibli R. (2015). Evaluation of the Radiolabeled Boronic Acid-Based FAP Inhibitor MIP-1232 for Atherosclerotic Plaque Imaging. Molecules.

[23] Trujillo-Benítez D., Luna-Gutiérrez M., Ferro-Flores G., Ocampo-García B., Santos-Cuevas C., Bravo-Villegas G., Morales-Ávila E., Cruz-Nova P., Díaz-Nieto L., García-Quiroz J. (2022). Design, Synthesis and Preclinical Assessment of ^99m^Tc-iFAP for In Vivo Fibroblast Activation Protein (FAP) Imaging. Molecules.

[24] Coria-Domínguez L., Vallejo-Armenta P., Luna-Gutiérrez M., Ocampo-García B., Gibbens-Bandala B., García-Pérez F., Ramírez-Nava G., Santos-Cuevas C., Ferro-Flores G. (2022). [^99m^Tc]Tc-iFAP Radioligand for SPECT/CT Imaging of the Tumor Microenvironment: Kinetics, Radiation Dosimetry, and Imaging in Patients. Pharmaceuticals.

[25] Lindner T., Loktev A., Altmann A., Giesel F., Kratochwil C., Debus J., Jäger D., Mier W., Haberkorn U. (2018). Development of Quinoline-Based Theranostic Ligands for the Targeting of Fibroblast Activation Protein. J. Nucl. Med..

[26] Loktev A., Lindner T., Burger E.-M., Altmann A., Giesel F., Kratochwil C., Debus J., Marme F., Jäger D., Mier W. (2019). Development of Fibroblast Activation Protein-Targeted Radiotracers with Improved Tumor Retention. J. Nucl. Med..

[27] Loktev A., Lindner T., Mier W., Debus J., Altmann A., Jäger D., Giesel F., Kratochwil C., Barthe P., Roumestand C. (2018). A Tumor-Imaging Method Targeting Cancer-Associated Fibroblasts. J. Nucl. Med..

[28] Giesel F.L., Kratochwil C., Lindner T., Marschalek M.M., Loktev A., Lehnert W., Debus J., Jäger D., Flechsig P., Altmann A. (2019). ^68^Ga-FAPI PET/CT: Biodistribution and Preliminary Dosimetry Estimate of 2 DOTA-Containing FAP-Targeting Agents in Patients with Various Cancers. J. Nucl. Med..

[29] Meyer C., Dahlbom M., Lindner T., Vauclin S., Mona C., Slavik R., Czernin J., Haberkorn U., Calais J. (2020). Radiation Dosimetry and Biodistribution of ^68^Ga-FAPI-46 PET Imaging in Cancer Patients. J. Nucl. Med..

[30] Lindner T., Altmann A., Kraemer S., Kleist C., Loktev A., Kratochwil C., Giesel F., Mier W., Marme F., Debus J. (2020). Design and Development of ^99m^Tc-Labeled FAPI Tracers for SPECT Imaging and ^188^Re Therapy. J. Nucl. Med..

[31] Hu K., Wang L., Wu H., Huang S., Tian Y., Wang Q., Xiao C., Han Y., Tang G. (2022). [^18^F]FAPI-42 PET Imaging in Cancer Patients: Optimal Acquisition Time, Biodistribution, and Comparison with [^68^Ga]Ga-FAPI-04. Eur. J. Nucl. Med. Mol. Imag..

[32] Giesel F.L., Adeberg S., Syed M., Lindner T., Jiménez-Franco L.D., Mavriopoulou E., Staudinger F., Tonndorf-Martini E., Regnery S., Rieken S. (2021). FAPI-74 PET/CT Using Either ^18^F-AlF or Cold-Kit ^68^Ga Labeling: Biodistribution, Radiation Dosimetry, and Tumor Delineation in Lung Cancer Patients. J. Nucl. Med..

[33] Hallett R.M., Poplawski S.E., Dornan M.H., Ahn S.H., Pan S., Wu W., Liu Y., Sanford D.G., Hergott V.S., Nguyen Q.-D. (2022). Pre-clinical Characterization of the Novel Fibroblast Activation Protein (FAP) Targeting Ligand PNT6555 for the Imaging and Therapy of Cancer. Cancer Res..

[34] Hallett R., Poplawski S., Novakowski K., Dornan M., Ahn S.H., Pan S., Wu W., Liu Y., Sanford D., Hergott V. (2022). Pre-clinical Characterization of the Novel FAP Targeting Ligand PNT6555 for the Imaging and Therapy of Cancer. J. Nucl. Med..

[35] FAPi Radioligand OpeN-Label, Phase 1 Study to Evaluate Safety, Tolerability and DosImetry of [Lu-177]-PNT6555; A Dose Escalation Study for TReatment of Patients with Select Solid Tumors (FRONTIER) (ClinicalTrials.gov Identifier: NCT05432193). NCT05432193.

[36] Toms J., Kogler J., Maschauer S., Daniel C., Schmidkonz C., Kuwert T., Prante O. (2020). Targeting Fibroblast Activation Protein: Radiosynthesis and Preclinical Evaluation of an ^18^F-Labeled FAP Inhibitor. J. Nucl. Med..

[37] Kelly J.M., Jeitner T.M., Ponnala S., Williams C., Nikolopoulou A., DiMagno S.G., Babich J.W. (2021). A Trifunctional Theranostic Ligand Targeting Fibroblast Activation Protein-α (FAPα). Mol. Imaging Biol..

[38] Tran E., Chinnasamy D., Yu Z., Morgan R.A., Lee C.-C.R., Restifo N.P., Rosenberg S.A. (2013). Immune Targeting of Fibroblast Activation Protein Triggers Recognition of Multipotent Bone Marrow Stromal Cells and Cachexia. J. Exp. Med..

[39] Kessler L., Ferdinandus J., Hirmas N., Zarrad F., Nader M., Kersting D., Weber M., Kazek S., Sraieb M., Hamacher R. (2022). Pitfalls and Common Findings in ^68^Ga-FAPI PET: A Pictorial Analysis. J. Nucl. Med..

[40] Wang S., Zhou X., Xu X., Ding J., Liu T., Jiang J., Li N., Zhu H., Yang Z. (2021). Dynamic PET/CT Imaging of ^68^Ga-FAPI-04 in Chinese Subjects. Front. Oncol..

[41] Bendre S., Zhang Z., Colpo N., Zeisler J., Wong A.A.W.L., Bénard F., Lin K.-S. (2023). Synthesis and Evaluation of ^68^Ga-Labeled (2*S*,4*S*)-4-Fluoropyrrolidine-2-Carbonitrile and (4*R*)-Thiazolidine-4-Carbonitrile Derivatives as Novel Fibroblast Activation Protein-Targeted PET Tracers for Cancer Imaging. Molecules.

[42] Coutts S.J., Kelly T.A., Snow R.J., Kennedy C.A., Barton R.W., Adams J., Krolikowski D.A., Freeman D.M., Campbell S.J., Ksiazek J.F. (1996). Structure−Activity Relationships of Boronic Acid Inhibitors of Dipeptidyl Peptidase IV. 1. Variation of the P_2_Position of X_aa_-boroPro Dipeptides. J. Med. Chem..

[43] Lin K.-S., Pan J., Amouroux G., Turashvili G., Mesak F., Hundal-Jabal N., Pourghiasian M., Lau J., Jenni S., Aparicio S. (2015). In Vivo Radioimaging of Bradykinin Receptor B1, a Widely Overexpressed Molecule in Human Cancer. Cancer Research.

[44] Verena A., Zhang Z., Kuo H.-T., Merkens H., Zeisler J., Wilson R., Bendre S., Wong A.A.W.L., Bénard F., Lin K.-S. (2023). Synthesis and Preclinical Evaluation of Three Novel ^68^Ga-Labeled Bispecific PSMA/FAP-Targeting Tracers for Prostate Cancer Imaging. Molecules.

[45] Bachovchin W.W., Lai H.S. (2007). Inhibitors of fibroblast activation protein alpha.

[46] Bu W., Akama T., Chanda S., Sullivan D., Ciaravino V., Jarnagin K., Freund Y., Sanders V., Chen C.-W., Fan X. (2012). Early Rapid Identification of in vivo Rat Metabolites of AN6414, a Novel Boron-containing PDE4 Inhibitor by QTRAP LC/MS/MS to Support Drug Discovery. J. Pharm. Biomed. Anal..

[47] Graham B.J., Windsor I.W., Gold B., Raines R.T. (2021). Boronic Acid with High Oxidative Stability and Utility in BiologicalC. Proc. Natl. Acad. Sci. USA.

[48] Marco-Dufort B., Tibbitt M.W. (2019). Design of Moldable Hydrogels for Biomedical Applications Using Dynamic Covalent Boronic Esters. Mater. Today Chem..

[49] Lau J., Rousseau E., Zhang Z., Uribe C.F., Kuo H.-T., Zeisler J., Zhang C., Kwon D., Lin K.-S., Bénard F. (2019). Positron Emission Tomography Imaging of the Gastrin-Releasing Peptide Receptor with a Novel Bombesin Analogue. ACS Omega.

