# Supplementary material for: Synthesis and Preclinical Evaluation of Novel 68Ga-Labeled (R)-Pyrrolidin-2-yl-boronic Acid-Based PET Tracers for Fibroblast Activation Protein-Targeted Cancer Imaging
Source: Pharmaceuticals (Basel). 2023 May 28;16(6):798. doi: 10.3390/ph16060798 (PMC10303363; doi:10.3390/ph16060798)
Supplement: Supplementary file 1 [file pharmaceuticals-16-00798-s001.zip › pharmaceuticals-2405921-supplementary.pdf]

Supplementary Materials

General Methods

The 2,3,5,6-tetrafluorophenyl 6-(3-(4-(*tert*-butoxycarbonyl)piperazin-1-yl)propoxy)quinoline-4-carboxylate (**1**), (*R*)-(1-glycylpyrrolidin-2-yl)boronic acid (1*S*,2*S*,3*R*,5*S*)-pinanediol ester, hydrochloride (**2**), (*R*)-(1-(D-alanyl)pyrrolidin-2-yl)boronic acid (1*S*,2*S*,3*R*,5*S*)-pinanediol ester, hydrochloride (**5**) and PNT6555 were prepared following the literature procedures [1–4]. All the other chemicals were procured from commercial sources and used without further purification. Flash column chromatography was carried out on a Teledyne ISCO CombiFlash NextGen 100 (Lincoln, NE) using RediSep gold silica gel flash columns (24 or 40 g silica prepacked, 20-40 µm particle size, 60 Å pore size). The purification and quality control of radiolabeling precursors, ^{nat}Ga-complexed standards and ^{68}Ga-labeled tracers were performed on (1) Agilent (Santa Clara, CA) high performance liquid chromatography (HPLC) systems equipped with a model 1200 quaternary pump, a model 1200 UV absorbance detector (set at 220 nm) and a Bioscan (Washington, DC) NaI scintillation detector, and (2) an Agilent 1260 Infinity II preparative system equipped with a model 1260 Infinity II preparative binary pump, a model 1260 Infinity variable wavelength detector (set at 220 nm) and a 1290 Infinity II preparative open-bed fraction collector. The operation of Agilent HPLC systems was controlled using the Agilent ChemStation software. The HPLC columns used were a semi-preparative column (Luna C18, 5 µm particle size, 100 Å pore size, 250 × 10 mm), an analytical column (Luna C18, 5 µm particle size, 100 Å pore size, 250 × 4.6 mm) from Phenomenex (Torrance, CA) and a preparative column (Gemini, NX-C18, 5 µm particle size, 110 Å pore size 50 mm × 30 mm). The collected HPLC eluates containing the desired products were lyophilized using a Labconco (Kansas City, MO) FreeZone 4.5 Plus freeze drier. The mass analyses were performed using a Waters (Milford, MA) Acquity QDa mass spectrometer with the equipped 2489 UV/Vis detector and e2695 Separations module. C18 Sep-Pak cartridges (1 cm^3, 50 mg) were obtained from Waters (Milford, MA). ^{68}Ga was eluted from an ITM Medical Isotopes GmbH (Munich, Germany) generator and purified according to the previously published procedures using a DGA resin column from Eichrom Technologies LLC (Lisle, IL) [5]. The radiolabeling of precursors was performed using Danby (Guelph, Canada) microwave oven model DMW7700WDB. The radioactivity of radiolabeled ligands was measured using a Capintec (Ramsey, NJ) CRC-25R/W dose calibrator. The radioactivity of mouse tissues collected from biodistribution studies was counted using a PerkinElmer (Waltham, MA) Wizard2 2480 automatic gamma counter.

Synthesis of DOTA-conjugated FAP-targeted ligands

Synthesis of (*R*)-(1-((6-(3-(4-(*tert*-butoxycarbonyl)piperazin-1-yl)propoxy)quinoline-4-carbonyl)glycyl)pyrrolidin-2-yl)boronic acid (1*S*,2*S*,3*R*,5*S*)-pinanediol ester (**3**)

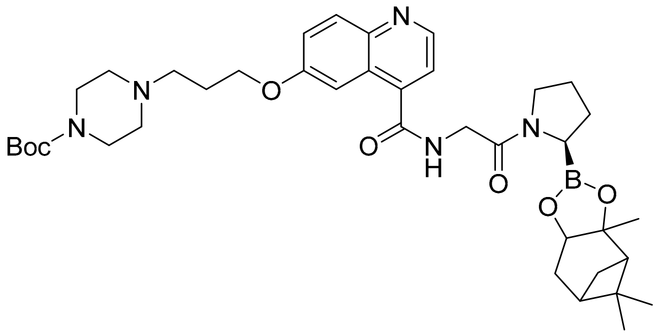

A mixture of compound **2** (215 mg, 0.63 mmol) and triethylamine (127 mg, 1.3 mmol, 175 µL) was dissolved in CH_3CN (5 mL). Compound **1** (311 mg, 0.55 mmol) was dissolved in CH_3CN (10 mL) and added to it. The resulting mixture was stirred at 80 °C for 2 days. After evaporation, the residue was purified by flash column chromatography (CombiFlash; 60 mL/min using 100% ethyl acetate as an eluent over 8 min followed by methanol/ethyl acetate gradient elution 0/100 to 20/80 over the next 12 min, t_R = 18.7 min) to obtain 137 mg (0.19 mmol, 35% yield) of compound **3** as a light purple solid**.** ESI-MS: calculated $[M+H]^+$ for $C_{38}H_{54}BN_5O_7$ 704.4; found 704.3. ^1H NMR (300 MHz, MeOD) δ 8.74 (s, 1H), 7.97 (d, *J* = 9.2 Hz, 1H), 7.76 (d, *J* = 2.7 Hz, 1H), 7.63 (d, *J* = 4.3 Hz, 1H), 7.46 (dd, *J* = 9.2, 2.7 Hz, 1H), 4.40 – 4.08 (m, 5H), 3.77 – 3.63 (m, 1H), 3.63 – 3.52 (m, 1H), 3.45 (t, *J* = 5.1 Hz, 5H), 3.31 – 3.11 (m, 1H), 2.63 (t, *J* = 7.6 Hz, 3H), 2.49 (q, *J* = 4.0 Hz, 5H), 2.44 – 2.27 (m, 1H), 2.23 – 2.01 (m, 5H), 2.05 – 1.91 (m, 1H), 1.91 – 1.81 (m, 1H), 1.86 – 1.69 (m, 1H), 1.46 (s, 9H), 1.41 – 1.36 (m, 2H), 1.33 (s, 1H), 1.26 (s, 2H), 0.98 – 0.82 (m, 1H), 0.84 (s, 2H).

Synthesis of the (1*S*,2*S*,3*R*,5*S*)-pinanediol ester of SB02055 (**4**)

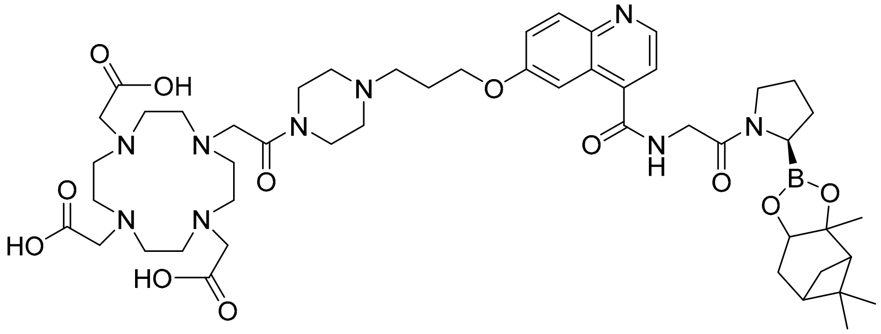

Boc-removal of compound **3** (12 mg, 17 µmol) was accomplished in 20 mL 1:1 (v/v) ether:4M HCl/dioxane by stirring overnight at room temperature. The reaction was evaporated in vacuo and redissolved in 3 mL H_2O. The resulting solution was neutralized by dropwise addition of triethylamine (~40 µL). DOTA-NHS (35 mg, 46 µmol) was added and the reaction was stirred overnight at 55 °C. The crude mixture was purified with HPLC (C18 prep column, 30 mL/min, 0-80% CH_3CN in H_2O (containing 0.1% formic acid), t_R = 5.2 min). The elution fractions containing the desired product were collected and lyophilized to give 1.9 mg (1.9 µmol, 11% yield) of compound **4** a white powder. ESI-MS: calculated $[M+H]^+$ $C_{49}H_{72}BN_9O_{12}$ 990.5; found 990.4.

Synthesis of the DOTA-conjugated precursor SB02055

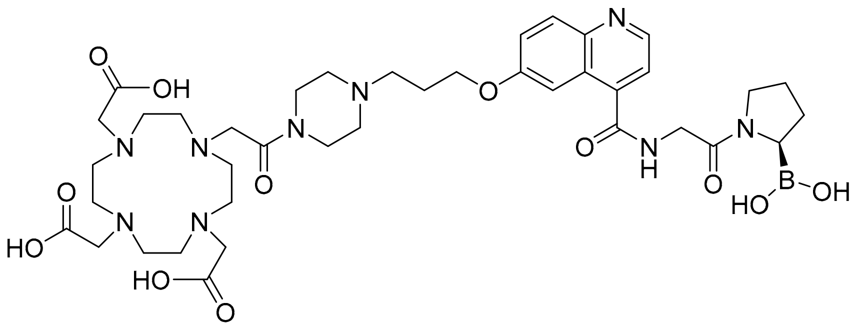

The pinanediol protecting group of compound **4** (1.9 mg, 1.9 µmol) was removed by treating it with a cleavage cocktail (4 mL) consisting of 95% TFA, 2.5% H_2O and 2.5% TIS for 4 h at room temperature. The reaction mixture was diluted with ether and evaporated to obtain a crude residue that was purified with HPLC (C18 semi-prep column, 4.5 mL/min, 3% CH_3CN in H_2O (containing 0.1% formic acid), t_R = 12.6 min). The elution fractions containing the desired product were collected and lyophilized to afford 0.9 mg, (1.1 µmol, 55% yield) of SB02055 as a white powder. ESI-MS: calculated $[M+H–H_2O]^+$ and $[M+Na–H_2O]^+$ for SB02055 ($C_{39}H_{58}BN_9O_{12}$) 838.4 and 860.4; found 838.2 and 860.5, respectively.

Synthesis of the ((*R*)-1-((6-(3-(4-(*tert*-butoxycarbonyl)piperazin-1-yl)propoxy)quinoline-4-carbonyl)-D-alanyl)pyrrolidin-2-yl)boronic acid (1*S*,2*S*,3*R*,5*S*)-pinanediol ester (**6**)

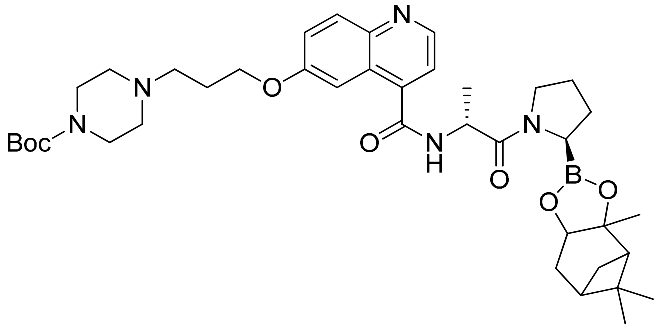

A mixture of compound **5** (101 mg, 0.28 mmol) and triethylamine (86 mg, 0.85 mmol, 118 µL) was dissolved in CH_3CN (5 mL). Compound **1** (123 mg, 0.22 mmol) was dissolved in CH_3CN (10 mL) and added to it. The resulting mixture was stirred at 80 °C for 2 days. After evaporation, the residue was purified by flash column chromatography (CombiFlash, 60 mL/min using 100% ethyl acetate as an eluent over 5 min followed by methanol/ethyl acetate gradient elution 0/100 to 15/85 over next 10 min, t_R = 12.1 min) to obtain 73 mg (0.10 mmol, 47% yield) of compound **6** as a light purple solid. ESI-MS: calculated $[M+H]^+$ for $C_{39}H_{56}BN_5O_7$ 718.4; found 718.4. ^1H NMR (300 MHz, MeOD) δ 8.75 (br, 1H), 7.99 (d, *J* = 9.2 Hz, 1H), 7.67 (d, *J* = 2.7 Hz, 1H), 7.59 (d, *J* = 4.3 Hz, 1H), 7.48 (dd, *J* = 9.2, 2.8 Hz, 1H), 5.01 (q, *J* = 7.0 Hz, 1H), 4.34 – 4.12 (m, 3H),

3.88 (dt, J = 10.6, 7.6 Hz, 1H), 3.69 (ddd, J = 10.2, 7.9, 3.6 Hz, 1H), 3.46 (t, J = 5.1 Hz, 4H), 3.08 (dd, J = 9.8, 6.9 Hz, 1H), 2.63 (t, J = 7.5 Hz, 3H), 2.53 – 2.44 (m, 4H), 2.37 – 2.14 (m, 1H), 2.14 – 1.99 (m, 4H), 1.98 – 1.84 (m, 1H), 1.84 – 1.72 (m, 2H), 1.48 (d, J = 7.0 Hz, 3H), 1.46 (s, 9H), 1.35 (s, 3H), 1.34 – 1.21 (m, 2H), 1.19 (s, 3H), 0.91 – 0.83 (m, 1H), 0.81 (s, 3H).

Synthesis of the (1*S*,2*S*,3*R*,5*S*)-pinanediol ester of SB04028 (**7**)

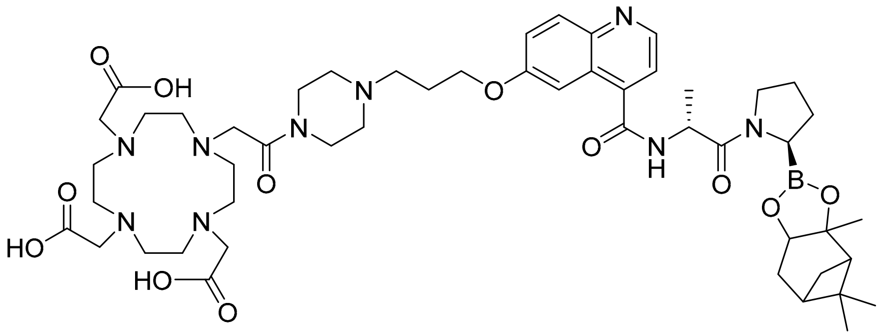

Boc-removal of compound **6** (24 mg, 33.7 µmol) was accomplished in 20 mL 1:1 (v/v) ether:4M HCl/dioxane by stirring overnight at room temperature. The reaction was evaporated in vacuo and redissolved in 3 mL H_2O. The resulting solution was neutralized by the dropwise addition of triethylamine (~35 µL). DOTA-NHS (39 mg, 51 µmol) was added and the reaction was stirred overnight at 55 °C. The crude mixture was purified with HPLC (C18 prep column, 30 mL/min, 0-80% CH_3CN in H_2O (containing 0.1% formic acid), t_R = 5.6 min). The elution fractions containing the desired product were collected and lyophilized to give 4.8 mg (4.8 µmol, 14% yield) of compound **7**, a white powder. ESI-MS: calculated $[M+H]^+$ for $C_{50}H_{74}BN_9O_{12}$ 1004.6; found 1004.4.

Synthesis of the DOTA-conjugated precursor SB04028

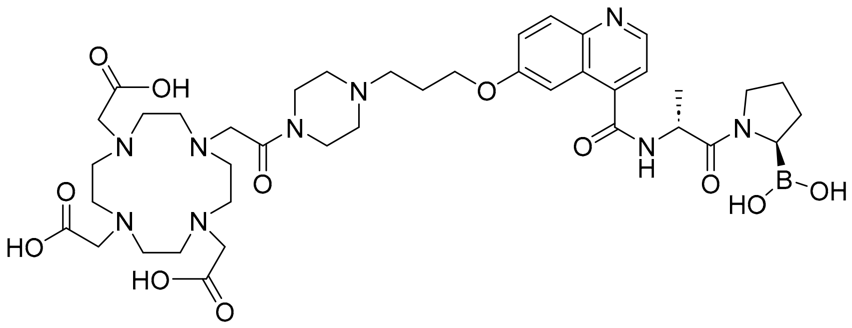

The pinanediol protecting group of compound **7** (4.8 mg, 4.8 µmol) was removed by treating it with a cleavage cocktail (4 mL) consisting of 95% TFA, 2.5% H_2O and 2.5% TIS for 4 h at room temperature. The reaction mixture was diluted with ether and evaporated to obtain a crude residue that was purified with HPLC (C18 semi-prep column, 4.5 mL/min, 9% CH_3CN in H_2O (containing 0.1% formic acid), t_R = 13.9 min). The elution fractions containing the desired

product were collected and lyophilized to afford 2.6 mg (3.0 µmol, 63% yield) of precursor SB04028 as a white powder. ESI-MS: calculated $[M+H–H_2O]^+$ and $[M+Na–H_2O]^+$ for SB04028 ($C_{40}H_{60}BN_9O_{12}$) 852.5 and 874.5; found 852.5 and 874.4, respectively.

General procedure for the synthesis of ^{nat}Ga-complexed standards

Following our previously published procedures [4], 2 mg of precursor was dissolved in 0.2 mL NaOAc buffer (0.1 N, pH 4.5) and $^{nat}GaCl_3$ (~5 eq., 42 µL, 0.27 M) was added. The reaction mixture was incubated at 90 °C for 30 min and then purified using HPLC. The elution fractions containing the desired product were collected and lyophilized to give a white powder.

Synthesis of the ^{nat}Ga-PNT6555

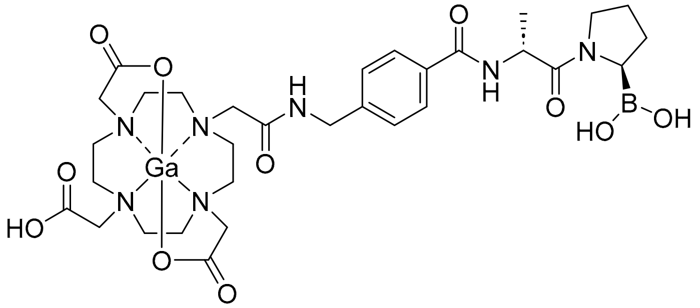

HPLC conditions: C18 semi-prep column, 4.5 mL/min, 13% CH_3CN in H_2O (containing 0.1% TFA), t_R = 12.1 min. Yield: 98%. ESI-MS: calculated $[M–H_2O]^+$ for ^{nat}Ga-PNT6555 $C_{31}H_{45}BGaN_7O_{11}$ 754.3; found 754.2.

Synthesis of the ^{nat}Ga-SB02055

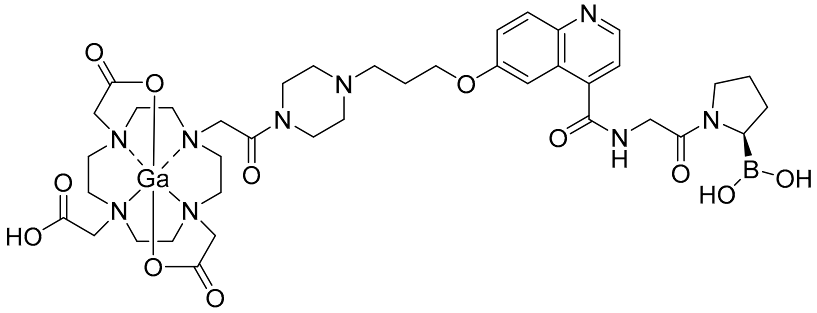

HPLC conditions: C18 semi-prep column, 4.5 mL/min, 5% CH_3CN in H_2O (containing 0.1% formic acid), t_R = 16.8 min. Yield: 75%. ESI-MS: calculated $[M+2H–H_2O]^{2+}$ for ^{nat}Ga-SB02055 ($C_{39}H_{55}BGaN_9O_{12}$) 453.2; found 453.2.

Synthesis of the ^{nat}Ga-SB04028

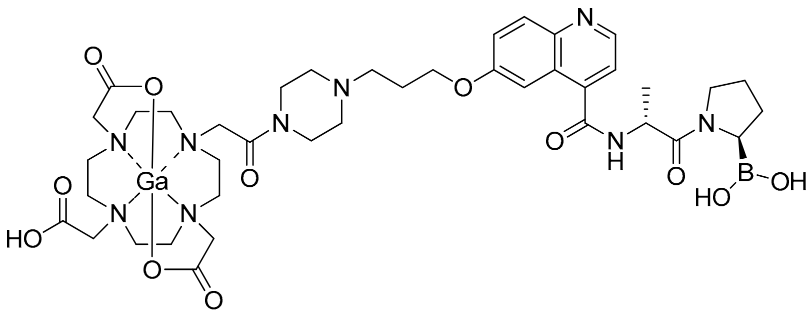

HPLC conditions: C18 semi-prep column, 4.5 mL/min, 10% CH_3CN in H_2O (containing 0.1% TFA), t_R = 11.3 min. Yield: 36%. ESI-MS: calculated $[M+2H–H_2O]^{2+}$ for Ga-SB04028 ($C_{40}H_{57}BGaN_9O_{12}$) 460.2; found 460.7.

General procedure for the synthesis of ^{68}Ga-complexed tracers

Following our previously published procedures [4], purified [^{68}Ga]$GaCl_3$ (158 to 396 MBq) in 0.55 mL water was added to a solution of 10 nmol precursor in 0.65 mL HEPES buffer (2M, pH 5.0). The reaction mixture was incubated in microwave oven for 1 min at power level 2. After cooling down for 1 min at an ambient temperature, the mixture was then purified using HPLC. The eluate fractions containing ^{68}Ga-labeled tracer were collected, diluted with DI water (50 mL) and passed through a C18 Sep-Pak cartridge. ^{68}Ga-labeled tracer trapped on the cartridge was eluted off with ethanol (containing 100 ppm ascorbic acid) and formulated with PBS (containing 100 ppm ascorbic acid). Quality control by HPLC was conducted before the animal studies were performed.

HPLC conditions for the preparation of [^{68}Ga]Ga-SB02055: C18 semi-prep column, eluted with 5% CH_3CN in H_2O (containing 0.1% formic acid) at a flow rate of 4.5 mL/min (t_R = 18.0 min). HPLC conditions for the quality control of [^{68}Ga]Ga-SB02055: C18 analytical column, eluted with 9% CH_3CN in H_2O (containing 0.1% formic acid) at a flow rate of 2.0 mL/min (t_R = 8.2 min).

HPLC conditions for the preparation of [^{68}Ga]Ga-SB04028: C18 semi-prep column, eluted with 12% CH_3CN in H_2O (containing 0.1% TFA) at a flow rate of 4.5 mL/min (t_R = 17.0 min). HPLC conditions for the quality control of [^{68}Ga]Ga-SB04028: C18 analytical column, eluted with 15% CH_3CN in H_2O (containing 0.1% TFA) at a flow rate of 2.0 mL/min (t_R = 8.5 min).

HPLC conditions for the preparation of [^{68}Ga]Ga-PNT6555: C18 semi-prep column, eluted with 14% CH_3CN in H_2O (containing 0.1% TFA) at a flow rate of 4.5 mL/min (t_R = 15.2 min). HPLC conditions for the quality control of [^{68}Ga]Ga-PNT6555: C18 analytical column, eluted with 17% CH_3CN in H_2O (containing 0.1% TFA) at a flow rate of 2.0 mL/min (t_R = 9.2 min).

^{68}Ga-labeled SB02055, SB04028 and PNT6555 were obtained in 19-58% decay-corrected radiochemical yield with >92% radiochemical purity and >9.1 GBq/μmol molar activity.

Table S1: Biodistribution and tumor/organ uptake ratios of [^{68}Ga]Ga-SB02055, [^{68}Ga]Ga-SB04028 and [^{68}Ga]Ga-PNT6555 in HEK239T:hFAP tumor-bearing mice. For blocking, [^{68}Ga]Ga-SB04028 was co-injected with FAPI-04 (0.5 mg per mouse).

Tissue	[^{68}Ga]Ga-SB02055	[^{68}Ga]Ga-SB04028	[^{68}Ga]Ga-PNT6555	[^{68}Ga]Ga-SB04028
(%ID/g)	(1 h, unblocked) *N*=4	(1 h, unblocked) *N*=4	(1 h, unblocked) *N*=4	(1h, blocked) *N*=4
**Blood**	3.13 ± 1.25	0.97 ± 0.03	0.60 ± 0.04	0.49 ± 0.13
**Fat**	0.16 ± 0.05	0.07 ± 0.01	0.06 ± 0.02	0.14 ± 0.13
**Testes**	0.26 ± 0.10	0.18 ± 0.02	0.16 ± 0.03	0.12 ± 0.01
**Small Intestine**	0.80 ± 0.29	0.34 ± 0.02	0.64 ± 0.10	0.34 ± 0.04
**Large Intestines**	0.56 ± 0.24	0.13 ± 0.03	0.11 ± 0.03	0.08 ± 0.03
**Stomach**	0.53 ± 0.36	0.06 ± 0.01	0.11 ± 0.05	0.03 ± 0.00
**Pancreas**	2.07 ± 0.83	0.18 ± 0.02	0.13 ± 0.02	0.09 ± 0.02
**Spleen**	0.85 ± 0.27	0.25 ± 0.04	0.21 ± 0.08	0.18 ± 0.02
**Adrenal Glands**	1.17 ± 1.13	0.14 ± 0.10	0.56 ± 0.89	0.09 ± 0.06
**Kidneys**	1.74 ± 0.95	2.10 ± 0.33	2.29 ± 0.43	2.53 ± 0.57
**Liver**	0.94 ± 0.27	0.32 ± 0.03	1.49 ± 0.11	0.30 ± 0.05
**Heart**	1.23 ± 0.48	0.24 ± 0.02	0.17 ± 0.01	0.11 ± 0.00
**Lungs**	1.20 ± 0.35	0.59 ± 0.04	0.42 ± 0.04	0.78 ± 0.37
**Tumor**	1.08 ± 0.37	10.1 ± 0.42	6.38 ± 0.45	0.30 ± 0.04
**Muscle**	0.63 ± 0.20	0.13 ± 0.01	0.12 ± 0.02	0.66 ± 0.12
**Bone**	1.14 ± 0.37	0.24 ± 0.09	0.45 ± 0.12	0.01 ± 0.00
**Brain**	0.05 ± 0.02	0.02 ± 0.00	0.02 ± 0.00	0.02 ± 0.00
**Thyroid**	1.49 ± 0.47	0.35 ± 0.00	0.26 ± 0.02	0.14 ± 0.01
**Tumor/Muscle**	1.72 ± 0.19	79.9 ± 7.50	52.6 ± 5.86	0.46 ± 0.06
**Tumor/Blood**	0.35 ± 0.03	10.4 ± 0.70	10.7 ± 1.29	0.63 ± 0.09
**Tumor/Kidney**	0.67 ± 0.15	4.89 ± 0.72	2.87 ± 0.59	0.12 ± 0.03
**Tumor/Bone**	0.95 ± 0.06	48.1 ± 20.5	14.9 ± 3.34	33.9 ± 13.3

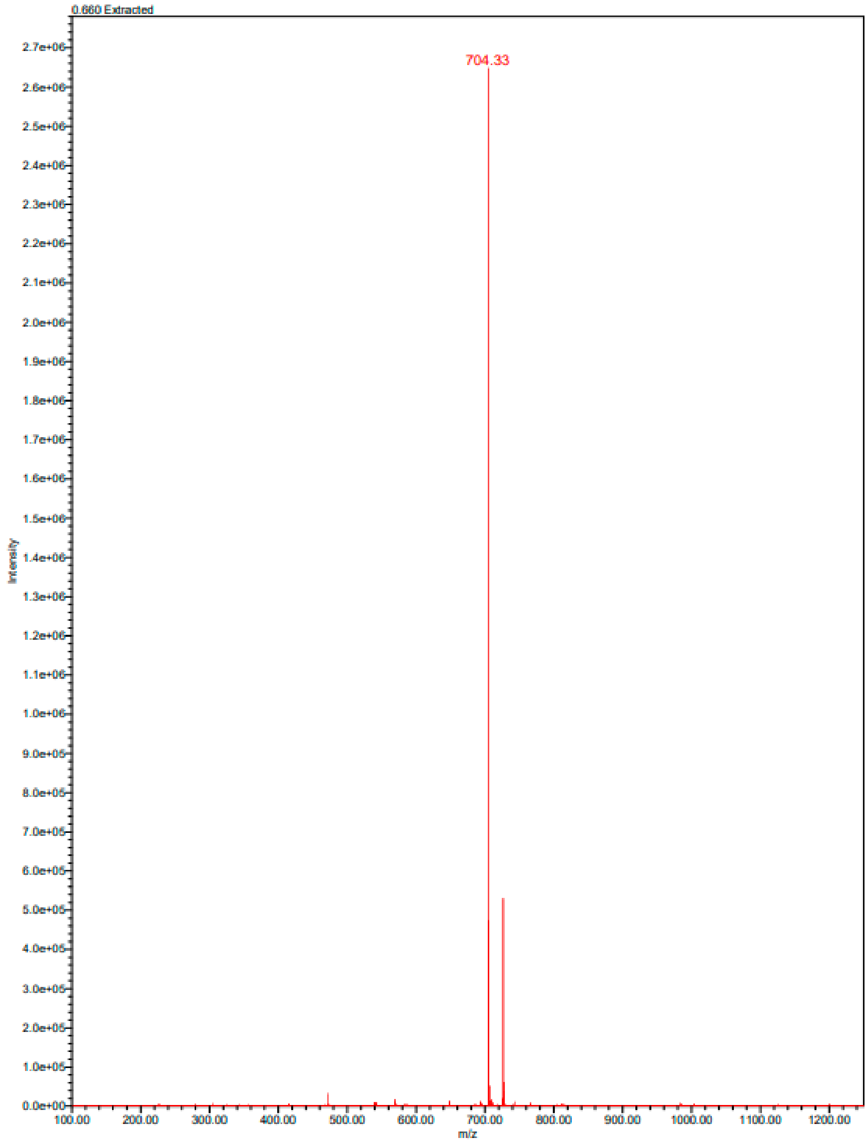

Figure S1: A representative MS spectrum of compound **3**. The calculated m/z value for $[M+H]^+$ is 704.4; observed 704.3.

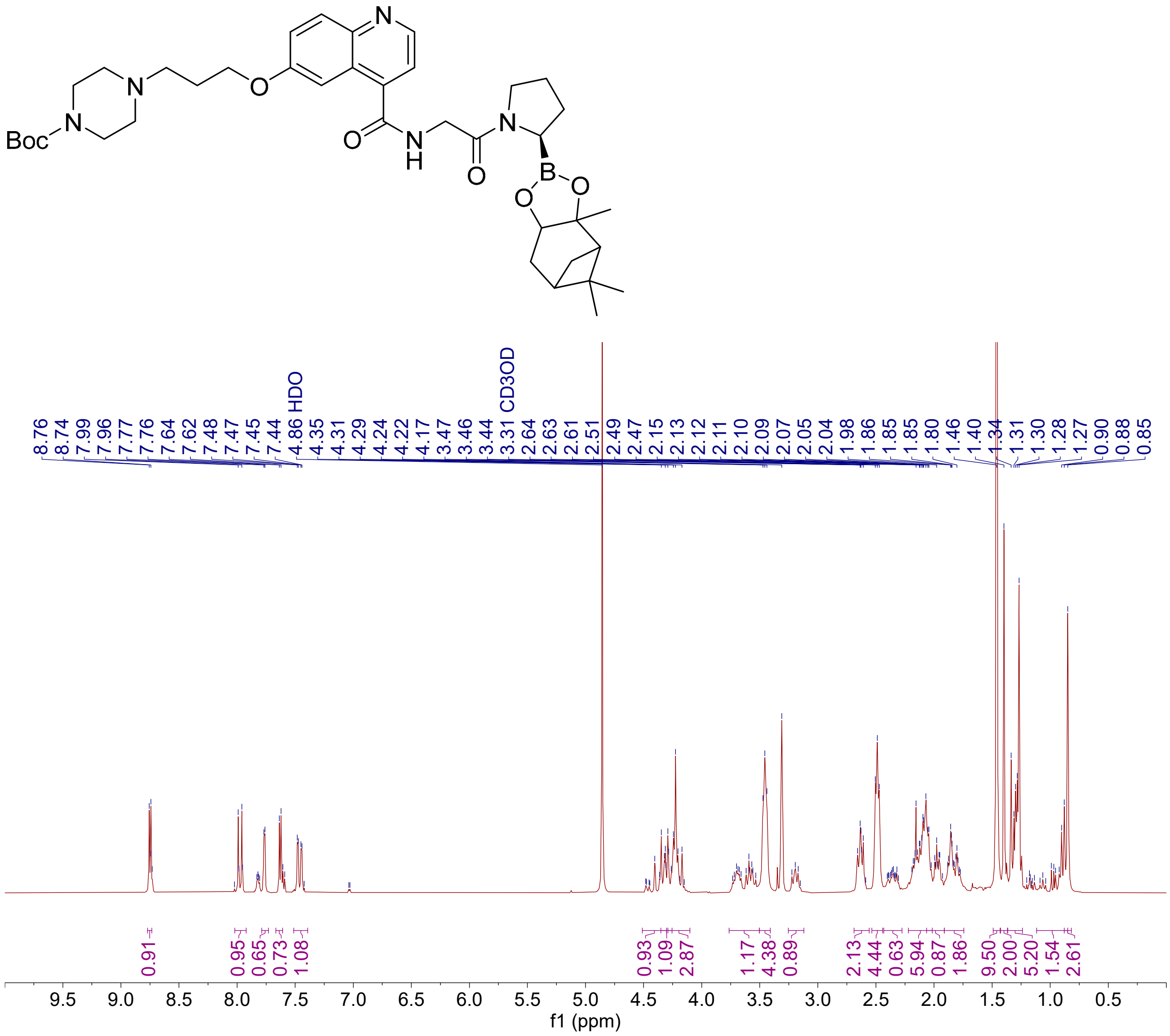

Figure S2: A representative NMR spectrum of compound **3**.

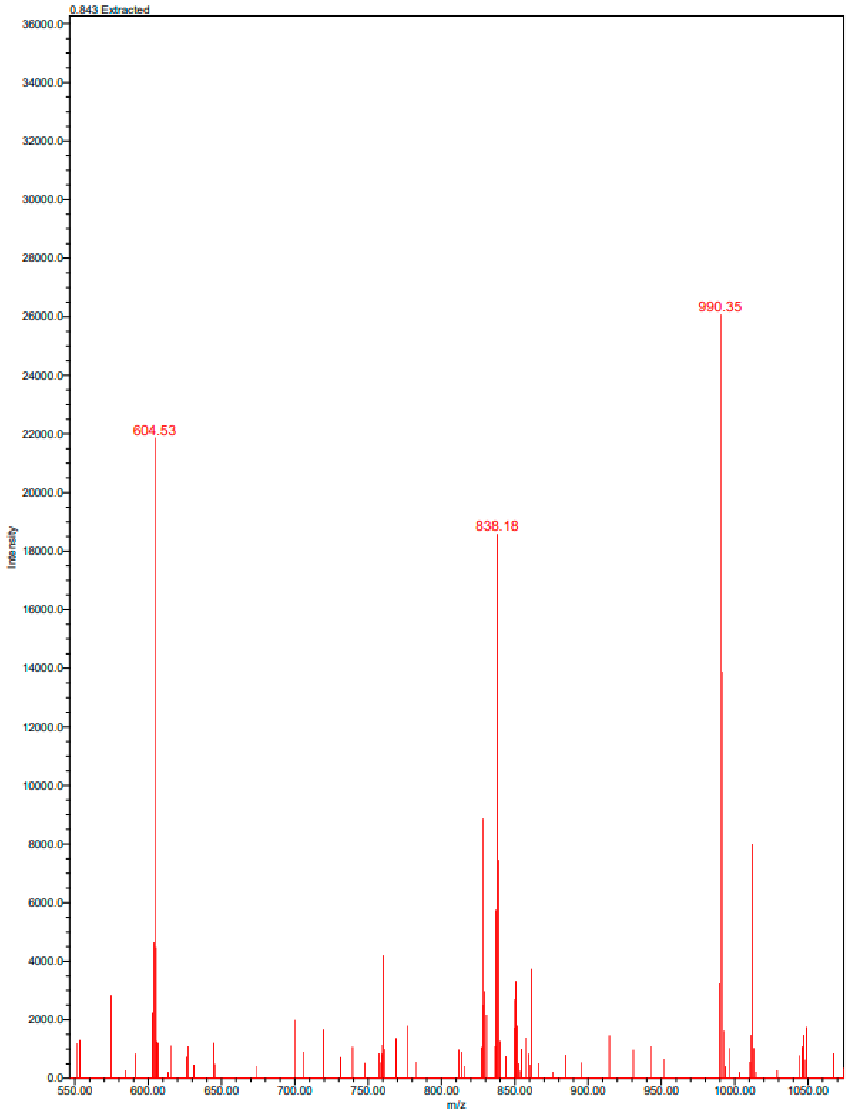

Figure S3: A representative MS spectrum of compound **4**. The calculated m/z value for $[M+H]^+$ is 990.5; observed 990.4.

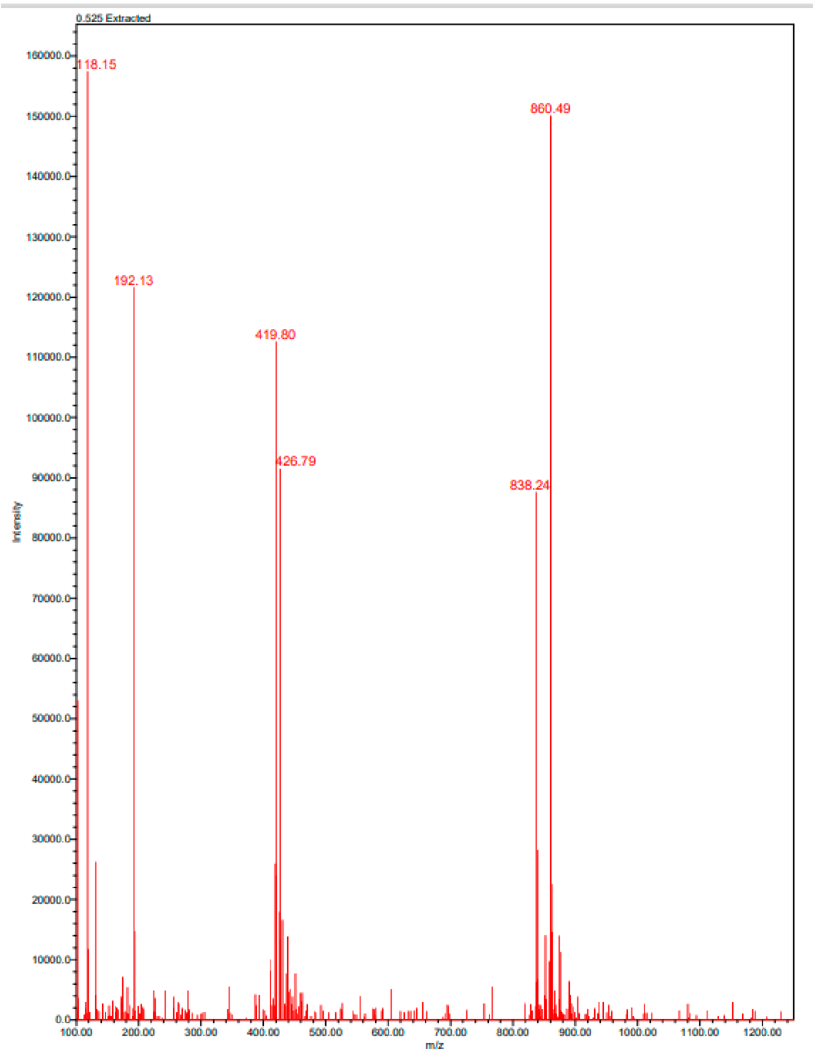

Figure S4: A representative MS spectrum of SB02055. The calculated m/z values for $[M+H–H_2O]^+$ and $[M+Na–H_2O]^+$ are 838.4 and 860.4, respectively; observed 838.2 and 860.5, respectively.

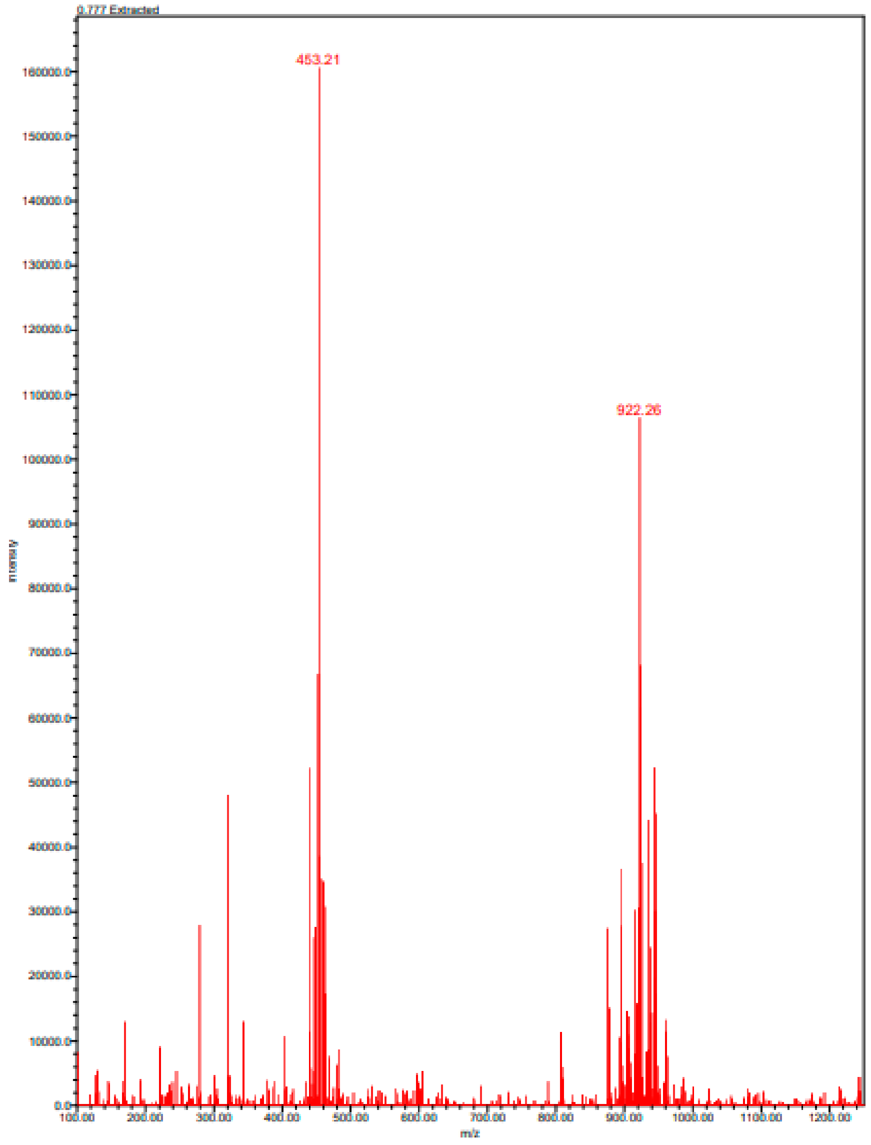

Figure S5: A representative MS spectrum of ^{nat}Ga-SB02055. The calculated m/z value for $[M+2H–H_2O]^{2+}$ is 453.2; observed 453.2.

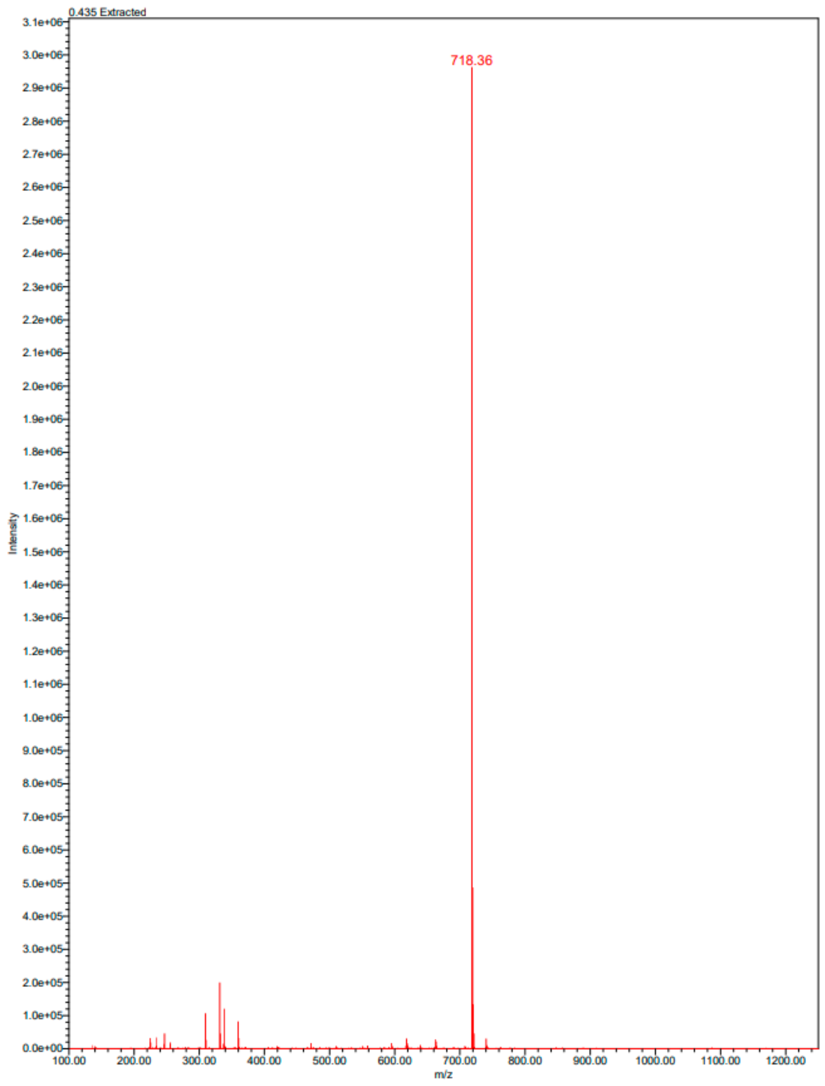

Figure S6: A representative MS spectrum of compound **6**. The calculated m/z value for $[M+H]^+$ is 718.4; observed 718.4.

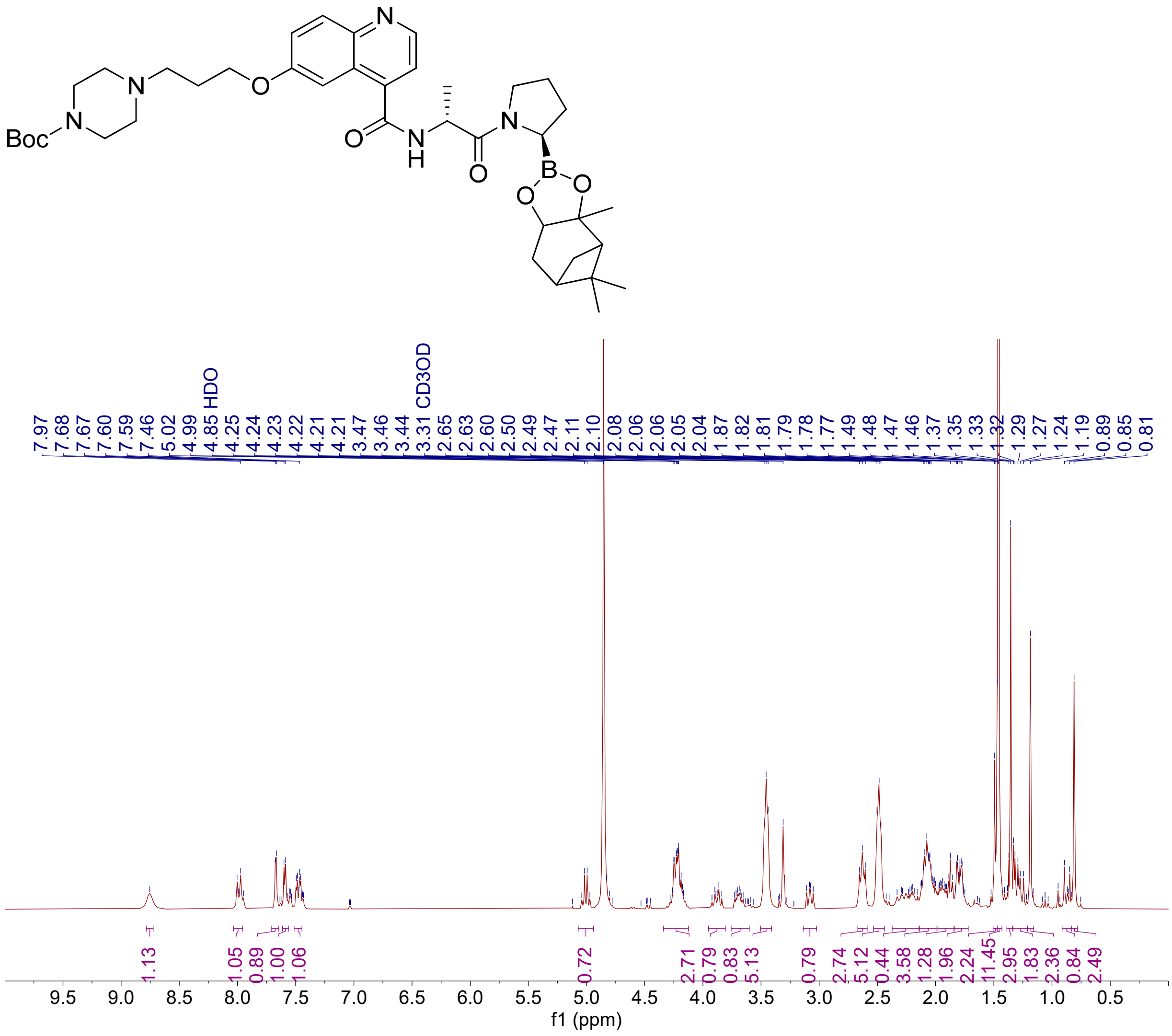

Figure S7: A representative NMR spectrum of compound **6**.

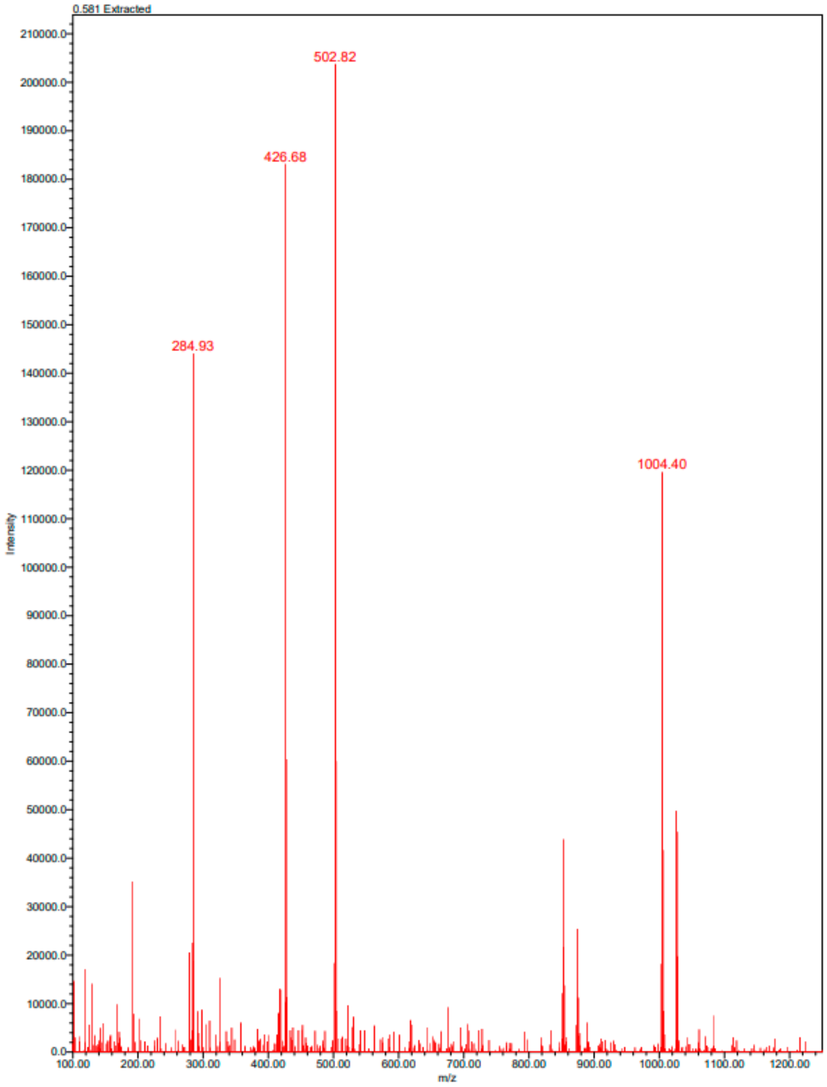

Figure S8: A representative MS spectrum of compound **7**. The calculated m/z value for $[M+H]^+$ is 1004.6; observed 1004.4.

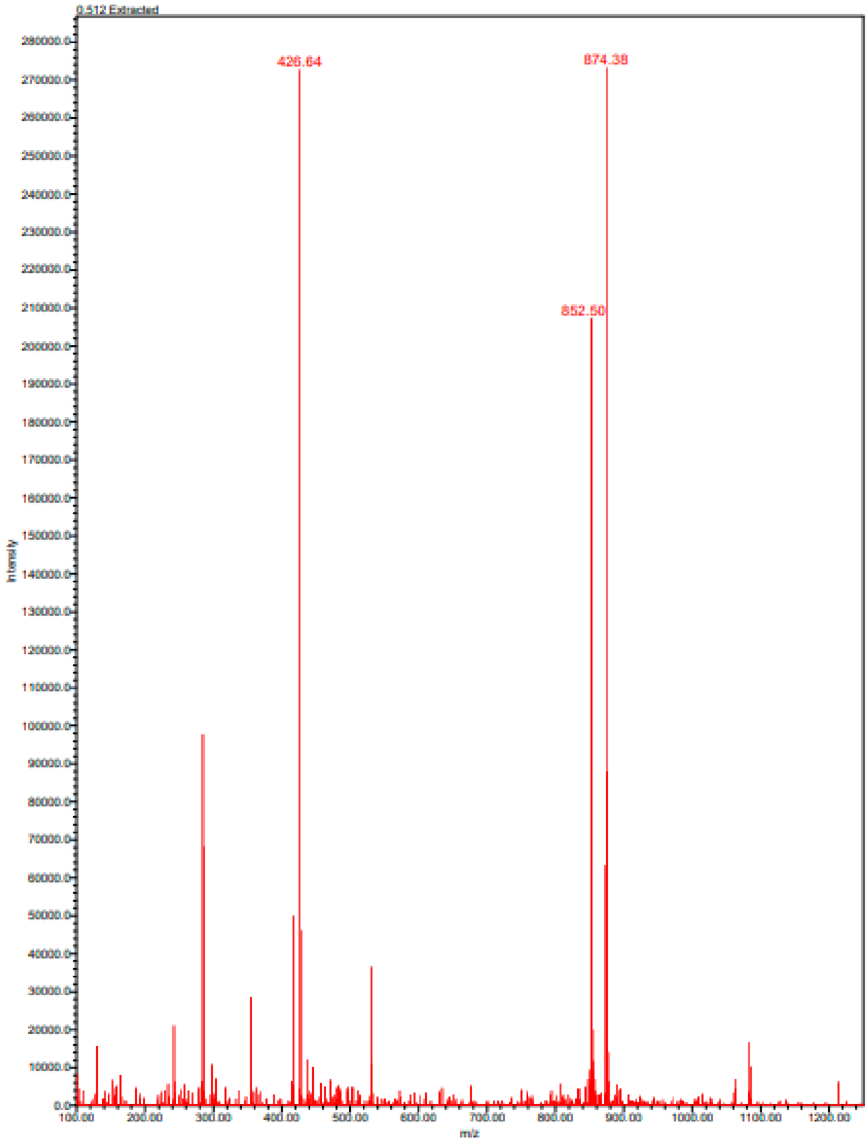

Figure S9: A representative MS spectrum of SB04028. The calculated m/z values for $[M+H–H_2O]^+$ and $[M+Na–H_2O]^+$ are 852.5 and 874.5, respectively; observed 852.5 and 874.4, respectively.

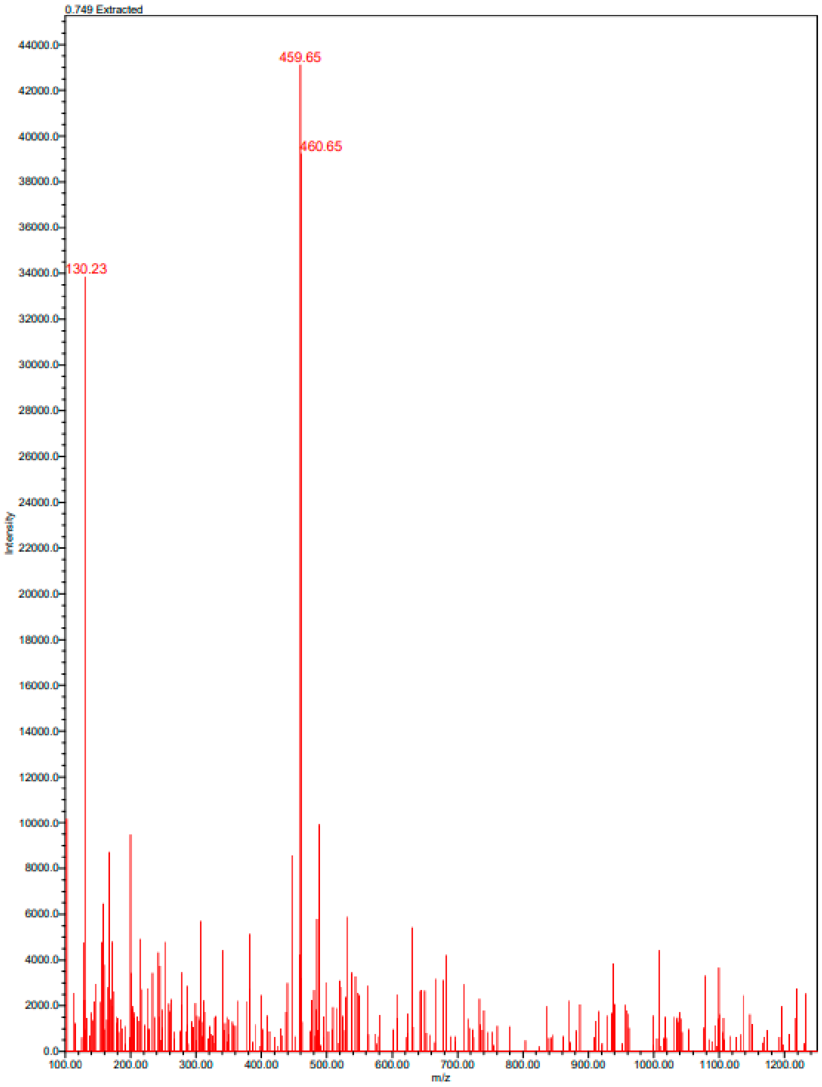

Figure S10: A representative MS spectrum of ^{nat}Ga-SB04028. The calculated m/z value for $[M+2H–H_2O]^+$ is 460.2; observed 460.7.

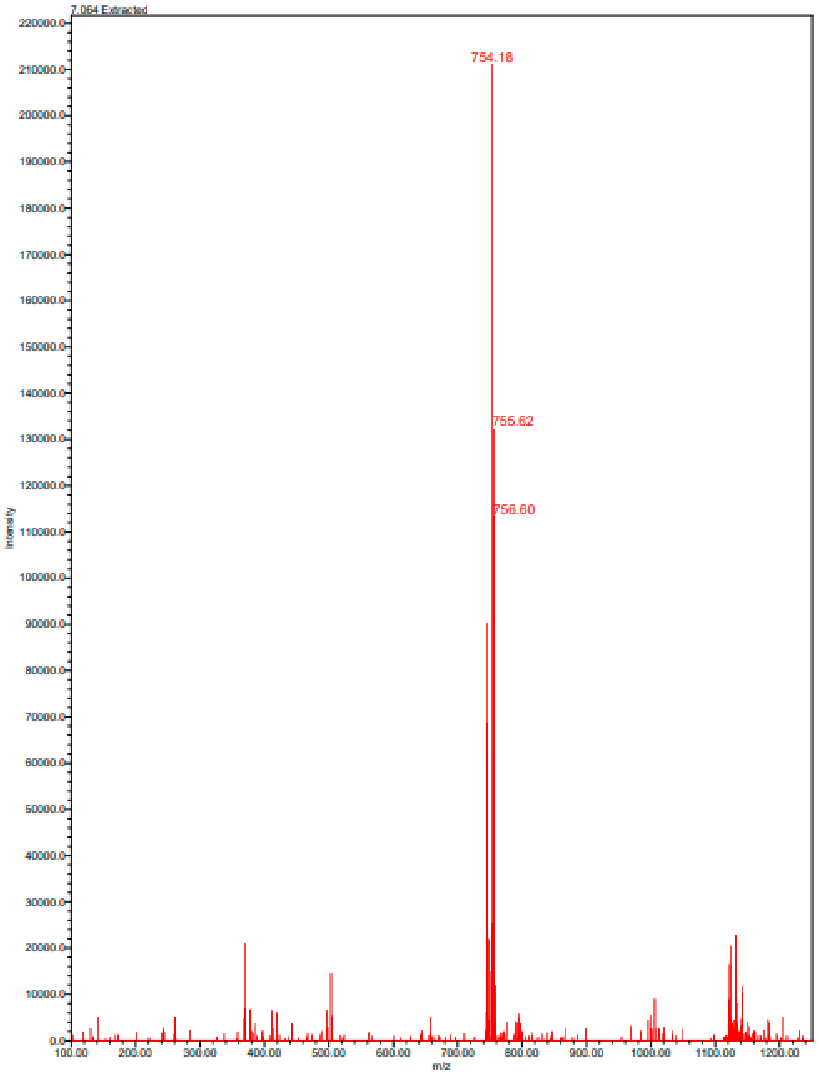

Figure S11: A representative MS spectrum of ^{nat}Ga-PNT6555. The calculated m/z value for $[M-H_2O]^+$ is 754.3; observed 754.2.

(A)

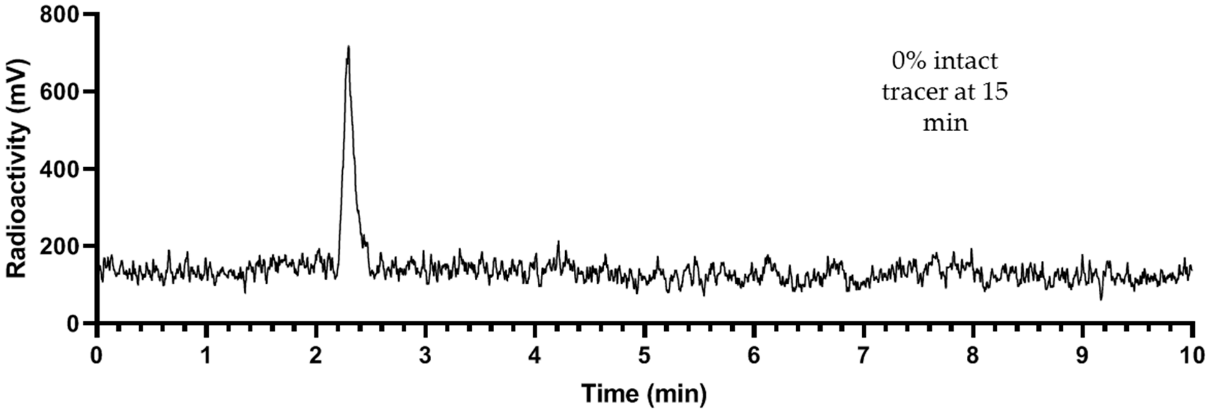

(B)

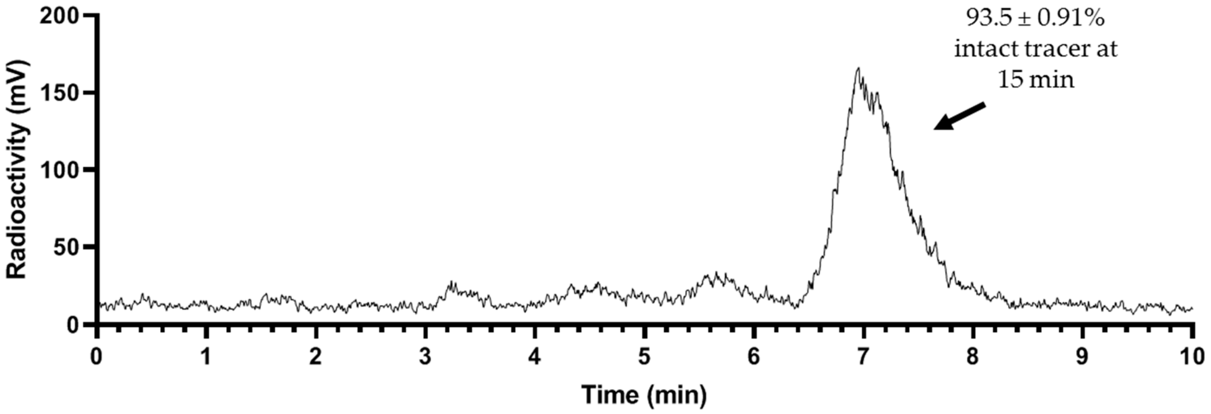

Figure S12: Representative radio-HPLC chromatograms from in an vivo stability study performed to determine the percent of the intact fraction of [^{68}Ga]Ga-SB02055 in mouse plasma (A) and urine (B) samples collected at 15 min post-injection. The peak pointed by an arrow is the intact tracer.

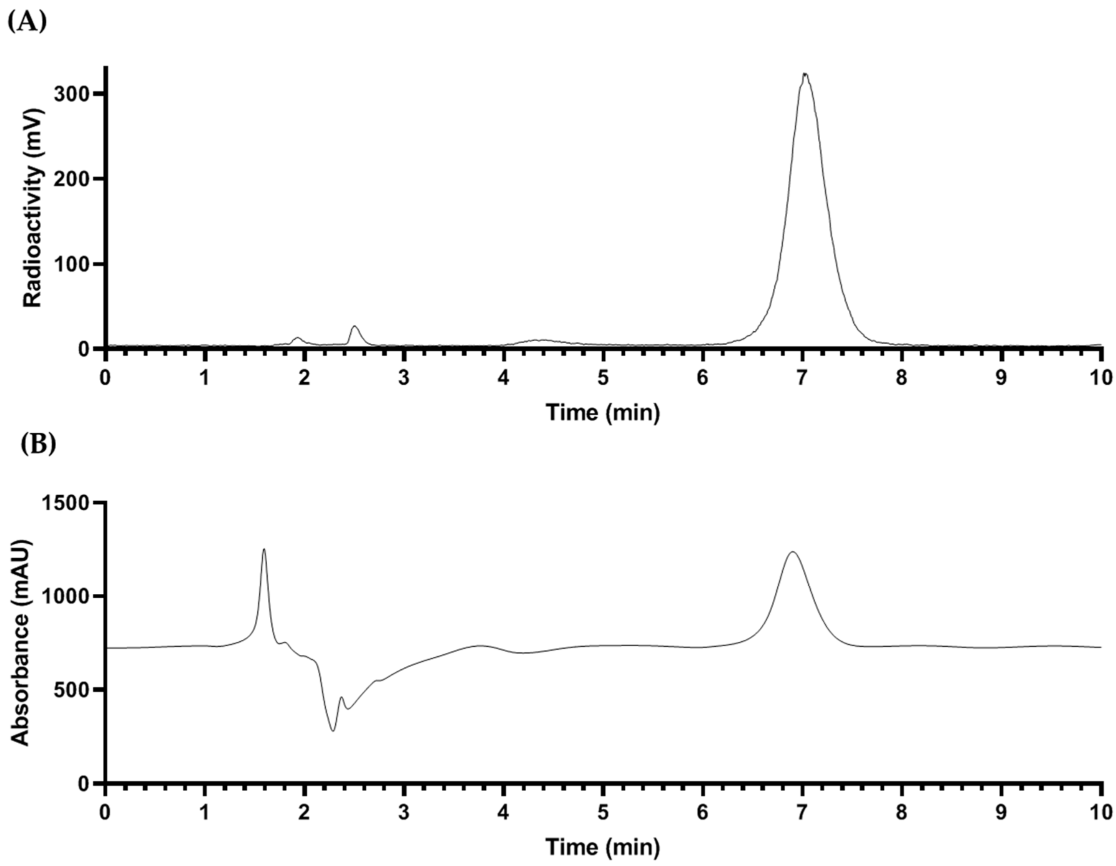

Figure S13: Radio-HPLC analysis of [^{68}Ga]Ga-SB02055 (A) QC radio-chromatogram (B) UV-chromatogram upon co-injection of ^{nat}Ga-SB02055.

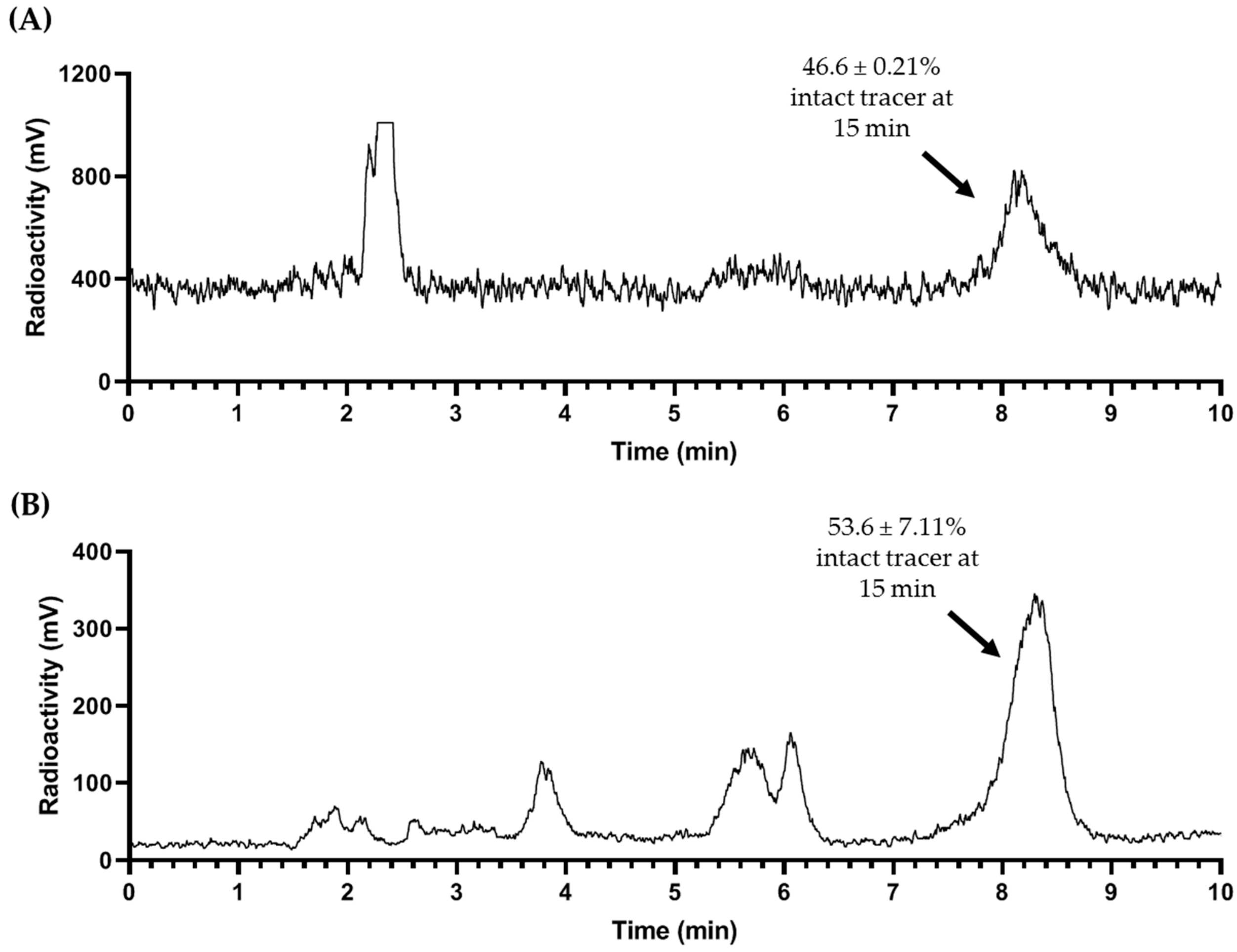

Figure S14: Representative radio-HPLC chromatograms from in an vivo stability study performed to determine the percent of the intact fraction of [^{68}Ga]Ga-SB04028 in mouse plasma (A) and urine (B) samples collected at 15 min post-injection. The peak pointed by an arrow is the intact tracer.

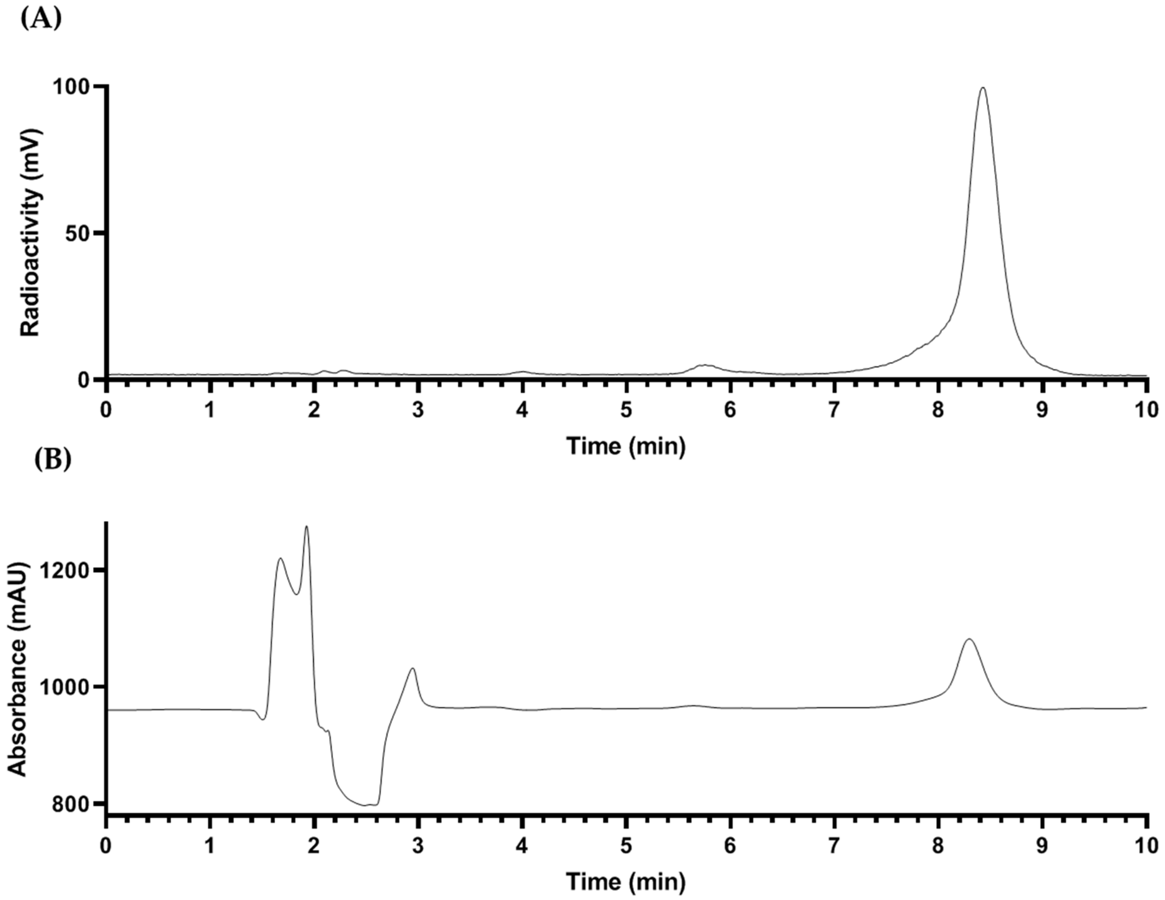

Figure S15: Radio-HPLC analysis of [^{68}Ga]Ga-SB04028 (A) QC radio-chromatogram (B) UV-chromatogram upon co-injection of ^{nat}Ga-SB04028.
